# TRV130 inhibits colon cancer progression via suppressing the Hedgehog signaling pathway: in vitro and in vivo evidence

**DOI:** 10.1186/s41065-026-00633-6

**Published:** 2026-01-19

**Authors:** Yuanzhao Zhuang, Changcheng Jiang, Yuqing Guo, Jiaxiao Sun

**Affiliations:** Department of Anesthesiology, Quanzhou first hospital Affiliated to Fujian Medical University, No. 250, Dong Street, Licheng District, Quanzhou, Fujian Province 515041 China

**Keywords:** Colon cancer, Oliceridine, Hedgehog, Proliferation, Apoptosis

## Abstract

**Background:**

Colon cancer, characterized by high incidence and mortality, faces clinical challenges due to high recurrence rates and drug resistance. Aberrant activation of Hedgehog (Hh) signaling pathway is a key driver of colon cancer progression, making it a promising therapeutic target; however, current Hh inhibitors are limited by resistance and adverse effects. Drug repurposing offers a strategic alternative to accelerate oncology drug development. Oliceridine (TRV130) is a clinically approved, selective µ-opioid receptor agonist with a well-established safety profile for pain management. Notably, its potential anti-tumor activity and impact on oncogenic pathways like Hh signaling remain entirely unexplored. This study aims to investigate the anti-colon cancer efficacy of TRV130 and its underlying mechanisms, focusing on the Hh pathway, thereby evaluating its repurposing potential.

**Methods:**

To establish the rationale for targeting the Hh pathway and the novelty of investigating TRV130, a focused literature review was conducted using PubMed and Web of Science databases (search period: 2000–2023), employing keywords including “colon cancer,” “Hedgehog pathway,” “drug repurposing,” and “TRV130/Oliceridine.” Based on this foundational evidence, the anti-tumor effects of TRV130 were systematically evaluated. Cell proliferation was assessed via MTT, EdU, and colony formation assays. Apoptosis was evaluated by flow cytometry and TUNEL assays. Migration and invasion were analyzed by Transwell and wound healing assays. The expressions of Cyclin D1, Bcl-2, Caspase 3, and Hh pathway proteins (PTCH1, GLI1) were detected by Western blot. A subcutaneous xenograft model was established in nude mice using HCT116 cells to validate in vivo efficacy, with tumor tissues analyzed by immunohistochemistry (IHC) and Western blot.

**Results:**

TRV130 exhibited potent, dose-dependent inhibition of colon cancer cell proliferation, migration, and invasion. It induced apoptosis through both intrinsic and extrinsic pathways, as evidenced by the downregulation of Cyclin D1 and Bcl-2, and the upregulation of cleaved caspase 3. Mechanistically, TRV130 significantly suppressed the Hh signaling pathway, reducing the expression of its key effectors (GLI1 and PTCH1) to an extent comparable to cyclopamine (Cyc), a canonical Hh inhibitor. In vivo, TRV130 administration dose-dependently inhibited tumor growth in xenograft models, reducing both tumor volume and weight. IHC and Western blot analyses of tumor tissues confirmed the downregulation of Hh pathway proteins and pro-proliferative markers, alongside upregulation of apoptotic markers.

**Conclusion:**

This study identifies TRV130 as a novel inhibitor of the Hh pathway, demonstrating its significant anti-colon cancer effects in vitro and in vivo. These findings reveal a previously unexplored, oncological mechanism for this clinically safe drug and support its repurposing as a promising therapeutic candidate, potentially offering an alternative to current Hh-targeted therapies. This work provides the foundational evidence for further development of TRV130 against colon cancer.

**Supplementary Information:**

The online version contains supplementary material available at 10.1186/s41065-026-00633-6.

## Introduction

Colon cancer is a highly prevalent gastrointestinal malignancy worldwide, and its incidence continues to rise [1]. Most patients are diagnosed at advanced stages, accompanied by a risk of recurrence and distant metastasis [2, 3]. Currently, clinical regimens combining surgery with chemoradiotherapy and targeted therapy can prolong the survival of some patients; however, challenges including chemoresistance, postoperative recurrence, and severe adverse reactions still significantly limit improvements in prognosis [4–6]. Therefore, exploring novel therapeutic strategies with both efficacy and safety, and expanding the therapeutic potential of approved drugs, has become a key direction for breaking through the bottlenecks in colon cancer treatment.

In recent years, the development of natural product-derived extracts, plant-derived nanoparticles, and novel targeted formulations has also provided diverse insights for cancer therapy [7–10]. Concurrently, the strategy of drug repurposing, which identifies new therapeutic uses for approved drugs, has gained prominence for its potential to accelerate clinical translation by leveraging existing safety and pharmacokinetic data. In parallel, advances in green nanotechnology, such as the use of plant-derived silver nanoparticles as potent radiosensitizers or magnetic nanocomposites for targeted drug delivery, underscore the ongoing pursuit of safer, more sustainable anticancer platforms [11–15]. Currently, the use of small-molecule drugs targeting G protein-coupled receptors (GPCRs) is an important strategy for cancer treatment. Research on GPCR-targeted drugs has made considerable progress [16, 17]. For instance, targeting adrenergic receptor pathways has shown promise in modulating tumor biology in cancers such as glioma [18], highlighting the broad therapeutic potential of modulating GPCR signaling in oncology. Oliceridine (TRV130) is a clinically approved biased µ-opioid receptor agonist (a class of GPCR-targeted small-molecules) used for treating moderate to severe acute pain. By selectively activating G protein signaling pathways and attenuating β-arrestin-mediated signal transduction, it retains potent analgesic effects while markedly reducing adverse reactions such as respiratory depression and gastrointestinal symptoms associated with traditional opioid drugs [19, 20]. To date, the clinical application of TRV130 has covered multiple settings including postoperative pain and traumatic pain, and its pharmacokinetic stability and safety are well-documented [21]. However, no studies have investigated whether TRV130 possesses anti-tumor activity, especially its effect on colon cancer and the associated molecular mechanisms. This represents a critical gap in exploring the expansion of its therapeutic utility.

To address this gap, it is critical to anchor the investigation in key signaling pathways that drive colon cancer progression, among which the Hedgehog (Hh) signaling pathway stands out as a well-validated core regulator of tumorigenesis and malignancy. It is primarily quiescent in normal adult tissues but is dysregulated and activated in a range of malignancies including colon cancer, with its activation level tightly linked to tumor malignancy grade and patient prognosis [22, 23]. Notably, while other oncogenic factors (e.g., AEG-1) and posttranscriptional events (e.g., alternative polyadenylation) contribute to colon cancer progression, the Hh pathway remains a well-validated core driver, making it a priority target for therapeutic intervention. The Hh pathway is activated when the transmembrane receptor patched 1 (PTCH1) relieves its inhibition of Smoothened (SMO); activated SMO then drives the transcription factor GLI family zinc finger 1 (GLI1), which in turn promotes proliferation, suppresses apoptosis, and enhances invasiveness [24–27]. Although studies have confirmed that targeting the Hh pathway can significantly attenuate the malignant phenotype of colon cancer, current clinically available pathway inhibitors still have issues such as high toxicity and susceptibility to acquired drug resistance [28]. Moreover, recent comprehensive analyses of colon adenocarcinoma have revealed that posttranscriptional regulatory events, such as alternative polyadenylation (APA), also contribute to tumor progression [29]. Specifically, dynamic APA changes in genes involved in cell-cycle regulation and extracellular matrix remodeling have been observed during tumorigenesis, which may alter gene expression patterns to promote cancer cell malignant transformation. These findings underscore the multi-layered complexity of colon cancer pathogenesis, further supporting the need for novel therapeutic agents that can target key signaling nodes with an improved safety and efficacy profile.

Based on this, this study systematically investigates the anti-colon cancer activity of TRV130, with a particular focus on clarifying its regulatory relationship with the Hh signaling pathway and the core underlying molecular mechanisms. This work aims to fill the research gap regarding the anti-tumor effect of TRV130, verify its clinical applicability for colon cancer treatment, and meanwhile provide a novel, safe, and controllable candidate drug for Hh pathway-targeted therapy, thereby laying an experimental foundation and providing theoretical support for breaking through the bottlenecks in colon cancer treatment.

## Materials and methods

### Cell lines

The human colon cancer cell lines Caco-2 and HCT116 were obtained from Wuhan Procell (Wuhan, China). HCT116 cells harbor a KRAS mutation (G13D) and wild-type p53, with low basal activity of the Hh signaling pathway but detectable expression of key signaling components (e.g., SMO, GLI1). Caco-2 cells are KRAS wild-type but carry a p53 mutation (R273H), exhibiting moderate baseline activation of the Hh pathway—likely linked to their differentiation status. Caco-2 cells were cultured in MEM containing non-essential amino acids (NEAA) (Procell, Wuhan, China, Cat. no. PM150410) supplemented with 20% fetal bovine serum (FBS) (Procell, Cat. no. 164210) and 1% penicillin-streptomycin (P/S) (Procell, Cat. no. PB180120). HCT116 cells were maintained in McCoy’s 5 A medium (Procell, Cat. no. PM150710) containing 10% FBS (Procell, Cat. no. 164210) and 1% P/S (Procell, Cat. no. PB180120). Both cell lines were incubated at 37℃ in a humidified atmosphere of 95% air and 5% CO_2_. Cells were treated with TRV130 (InvivoChem, Libertyville, IL, USA, Cat. no. V50861) at different concentrations (2.5, 5, 10, 20, 40, 80, and 160 µM) for 48 h in preliminary dose-response experiments. For subsequent experiments, cells were divided into four groups: control (Ctrl), 10 µM TRV130, 20 µM TRV130, and 40 µM TRV130. For subsequent experiments, cells were divided into four groups: Ctrl, 10 µM TRV130, 20 µM TRV130, and 40 µM TRV130. For experiments specifically focused on investigating the Hh signaling pathway, Caco-2 and HCT116 cells were also treated with 5 µM cyclopamine (Cyc; MedChemExpress, Monmouth Junction, NJ, USA), a positive control for Hh pathway inhibition.

### Cell viability assay

MTT Cell Proliferation and Cytotoxicity Assay Kit (Beyotime, Shanghai, China, Cat. no. C0009S) was employed to assess cell viability. 100 µL of cell suspension (1000 cells) was seeded into a 96-well plate for overnight incubation. After that, 10 µL of MTT solution was introduced, and the mixture was then incubated for an additional 4 h. Subsequently, 100 µL of formazan solubilization solution was added, and the incubation was continued until the formazan crystals were dissolved. Finally, the absorbance was measured at 570 nm.

### Cell proliferation detection

Cell proliferation was assessed using the BeyoClick™ EdU Cell Proliferation Kit with AF488 (Beyotime, Cat. no. C0071S). Prior to the experiment, the 2× EdU working solution was pre-warmed to 37℃ and added to wells at a 1:1 ratio with the existing culture medium. Following completion of EdU labeling, cells were sequentially fixed and permeabilized. The Click reaction solution was introduced, followed by incubation of the cells under dark conditions for 30 minutes. Nuclei were stained with 4’,6-Diamidino-2-Phenylindole, dihydrochloride (DAPI) (Beyotime), and EdU^+^ cells were counted to calculate the proliferation rate.

### Colony formation assay

Cells were seeded in 6-well plates (500 cells per well) and incubated at 37℃ for 14 days. When visible colonies formed, the medium was carefully removed, and cells were fixed with 4% paraformaldehyde (Sigma-Aldrich, St. Louis, MO, USA), followed by staining with 0.1% crystal violet solution (Beyotime). After the cells were thoroughly washed with phosphate-buffered saline (PBS), the plates were air-dried, and colonies were counted under a light microscope to quantify clonogenic potential.

### Flow cytometry

Apoptosis was evaluated using the Annexin V-FITC/PI Apoptosis Detection Kit (Yeasen, Shanghai, China, Cat. no. 40302ES50). Cells were digested and collected. The harvested cells were washed twice with pre-cooled PBS, yielding 5 × 10^5^ cells per sample. After removing PBS, cells were resuspended in 100 µL of 1× Binding Buffer, after which 5 µL Annexin V-FITC and 10 µL PI were added sequentially. The mixture was incubated for 15 min in the dark. Then, 400 µL of 1× Binding Buffer was added, and samples were analyzed using a flow cytometer within 1 h.

### Cell apoptosis assessment

Cell apoptosis was further evaluated using the CoraLite^®^594 TUNEL Apoptosis Detection Kit (Proteintech, Wuhan, China; Cat. no. PF00006). Cells were washed twice with PBS, followed by fixation and permeabilization. TUNEL reaction solution was prepared in strict accordance with the kit manufacturer’s protocols, with each sample receiving 50 µL of reaction solution containing 2 µL TdT enzyme. Cells were incubated at 37℃ for 60 min in the dark, after which the reaction solution was removed and the cells were rinsed with 1×PBS. Subsequently, cells were washed three times with a buffer consisting of 0.1% Triton X-100 and 5 mg/mL bovine serum albumin (BSA) prepared in PBS. Each sample was then treated with DAPI staining solution (2 µg/mL) for 10 min. Following staining, the samples were washed before analysis under a fluorescence microscope.

### Transwell assay

2 × 10^4^ cells suspended in serum-free medium were added to the upper chambers of 24-well Transwell inserts (8-µm pore size) (Corning, Tewksbury, MA, USA). For invasion detection, the upper chambers were pre-coated with Matrigel (1:8 dilution in serum-free medium) (Corning, Bedford, MA, USA) and incubated at 37℃ for 30 min to form a gel. The lower chambers were loaded with complete medium containing 10% FBS. Following a 24-hour incubation at 37℃, remaining non-migrated (or non-invaded) cells on the upper surface of the membrane were softly wiped off. Cells on the lower surface were fixed with 4% paraformaldehyde (Beyotime) for 15 min and stained with 0.1% crystal violet (Beyotime) for 20 min. Five random fields per insert were imaged under a light microscope, and cells were counted to quantify migration and invasion capacity.

### Wound healing assay

Cells were seeded in 6-well plates and cultured at 37℃ until reaching 90% confluence. A straight, consistent scratch was introduced into the cell monolayer via a sterile 200 µL pipette tip. Subsequent rinsing of the monolayer with serum-free medium (twice) eliminated detached cells and debris, after which the residual cells were incubated in fresh medium. Images of the wound area were captured at 0 h and 24 h using an inverted microscope. The wound width was measured at three random positions per image using ImageJ software, and the migration rate was calculated to quantify cell migration capacity.

### Western blot

Proteins were extracted from cells using RIPA Lysis and Extraction Buffer (Thermo Fisher Scientific, Waltham, MA, USA), and protein concentrations were determined by BCA assay. After electrophoresis, separated proteins were transferred to PVDF membranes (Millipore, Billerica, MA, USA), which were then blocked with 5% non-fat milk for 1 h at room temperature. Membranes were incubated with primary antibodies against Cyclin D1 (1:10000, Proteintech, Cat. no. 26939-1-AP), Bcl-2 (1:10000, Proteintech, Cat. no. 80313-1-RR), caspase 3 (1:800, Proteintech, Cat. no. 19677-1-AP), Cleaved-Caspase 3 (C-caspase 3; 1:1000, Proteintech, Cat. no. 25128-1-AP), β-actin (1:10000, Proteintech, Cat. no. 66009-1-Ig), GLI1 (1:20000, Proteintech, Cat. no. 66905-1-Ig), PTCH1 (1:1000, Cell Signaling Technology, Danvers, MA, USA, Cat. no. 2468 S), Bax (1:2000, Proteintech, Cat. no. 60267-1-Ig), Bim (1:500, Proteintech, Cat. no. 22037-1-AP), and FasL (1:1000, Cell Signaling Technology, Cat. no. 68405) overnight at 4℃, followed by HRP-conjugated anti-rabbit/mouse secondary antibodies (1:1000, Proteintech, Cat. no. SA00001-2/RGAM001) for 1 h at room temperature. After washing, protein bands were visualized using Clarity™ Western ECL Substrate Kit (Bio-Rad, Shanghai, China) and a chemiluminescence imaging system. Band intensities were quantified using ImageJ software and normalized to internal controls.

### Xenograft model

Female BALB/c nude mice (4–6 weeks old) were purchased from Beijing SPF Biotechnology Co., Ltd. (Beijing, China). For the establishment of subcutaneous colon cancer xenografts, HCT116 cells (5 × 10^6^ cells in 100 µL PBS) were subcutaneously injected into the right dorsal flank of each nude mouse. 14 days after cell inoculation, the in vivo TRV130 doses (1.18 mg/kg and 3.6 mg/kg) were selected based on PK/PD integration analysis (1.18 mg/kg as the minimum effective dose, 3.6 mg/kg achieving clinically equivalent exposure) and dose-escalation pilot experiments (no severe toxicity observed), with their converted human equivalent doses overlapping the clinical Phase I range to ensure clinical relevance. Mice were randomly divided into 4 groups (*n* = 5/group): control group (intraperitoneal injection of normal saline), TRV130 low-dose group (L, 1.18 mg/kg, intraperitoneal injection), TRV130 high-dose group (H, 3.6 mg/kg, intraperitoneal injection), and Cyc group (30 mg/kg, intraperitoneal injection). Administration was performed once daily for 25 consecutive days. Mouse body weight and tumor volume (V = 0.5 × length × width^2^, measured with a vernier caliper) were recorded every 5 days. Upon completion of the experiment, mice were euthanized, and tumors were dissected and weighed to evaluate the anti-tumor efficacy of TRV130. All animal procedures were approved by the Institutional Animal Care and Use Committee (IACUC) of Quanzhou first hospital Affiliated to Fujian Medical University and strictly followed the 3R principles of animal welfare.

### Immunohistochemistry (IHC)

IHC was performed on paraffin-embedded tumor tissue Sect. (4-µm thick). Sections were deparaffinized in xylene and rehydrated through a graded ethanol series, after which antigen retrieval was conducted in citrate buffer (pH 6.0) with microwave heating. Endogenous peroxidase activity was blocked with 3% H_2_O_2_ in methanol for 10 minutes, and nonspecific binding was inhibited by incubating sections with 5% BSA at room temperature for 1 hour. Sections were then incubated with anti-GLI1 (Proteintech, Cat. no. 66905-1-Ig) and anti-PTCH1 (Cell Signaling Technology, Cat. no. 2468S) overnight at 4℃, followed by HRP-conjugated secondary antibodies (Proteintech, Cat. no. SA00001-2/RGAM001) for 1 hour at room temperature. After washing with PBS, immunoreactivity was visualized using 3,3’-diaminobenzidine (DAB) substrate, and sections were counterstained with hematoxylin to label nuclei. Finally, sections were dehydrated, cleared in xylene, mounted with neutral mounting medium, and observed under a light microscope.

### Statistical analysis

Statistical analyses were performed using GraphPad Prism 9.0 software (GraphPad Software, Inc., USA), and data were presented as mean ± standard deviation (SD). One-way analysis of variance (one-way ANOVA) was applied to compare differences among multiple groups with a single independent variable. Two-way ANOVA was used to analyze data involving two independent variables and their interaction. A *P* value < 0.05 was considered statistically significant.

## Results

### TRV130 suppresses colon cancer cell proliferation

**Fig. 1:**
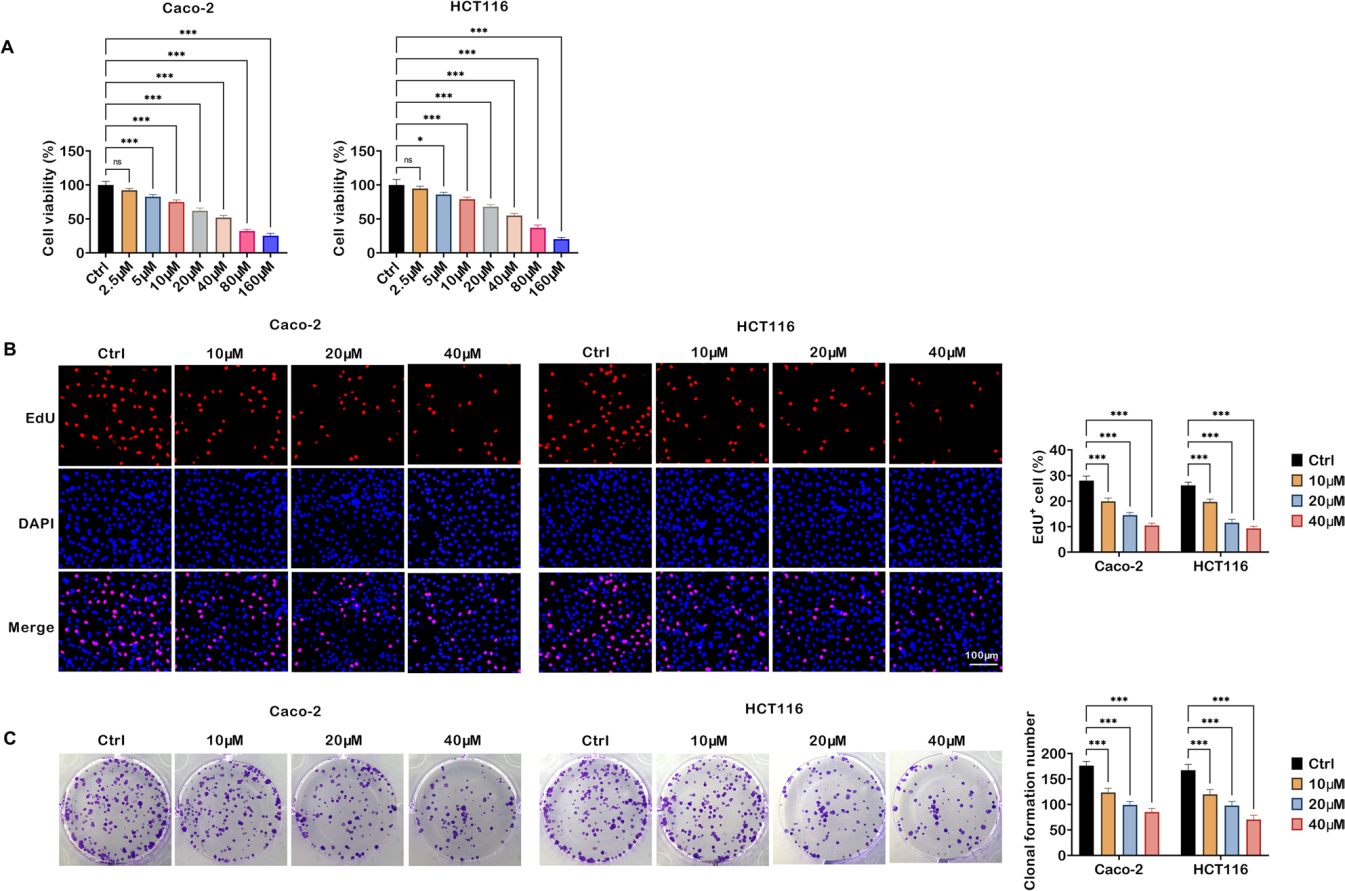
TRV130 represses proliferation of colon cancer cells. (**A**) Caco-2 and HCT116 cells were treated with TRV130 at varying concentrations (2.5, 5, 10, 20, 40, 80, and 160 µM) for 24 h, and cell viability was measured by MTT assay. (B-C) Caco-2 and HCT116 cells were treated with different concentrations of TRV130 (10, 20, and 40 µM). (**B**) EdU incorporation assay was used to evaluate cell proliferation in Caco-2 and HCT116 cells (Scale bar = 100 μm). (**C**) Colony formation assay was performed to assess the clonogenic potential of Caco-2 and HCT116 cells. * *P* < 0.05, *** *P* < 0.001, ns, not significant

To explore the impacts of TRV130 on the proliferative activity of colon cancer cells, a series of experiments were performed in Caco-2 and HCT116 cells. As shown in Fig. 1A, after 24-hour treatment with TRV130 at varying concentrations (2.5, 5, 10, 20, 40, 80, and 160 µM), the viability of colon cancer cells was significantly decreased in a concentration-dependent manner. To further assess cell proliferation, EdU incorporation assays were conducted. Relative to the Ctrl group, the proportion of EdU-positive cells in Caco-2 and HCT116 cells remarkably decreased as TRV130 concentrations increased (Fig. 1B). Colony formation assay revealed that TRV130 treatment reduced the number of colonies formed by Caco-2 and HCT116 cells (Fig. 1C). Collectively, TRV130 exerted a suppressive effect on colon cancer cell proliferation.

### TRV130 induces apoptosis in colon cancer cells

**Fig. 2:**
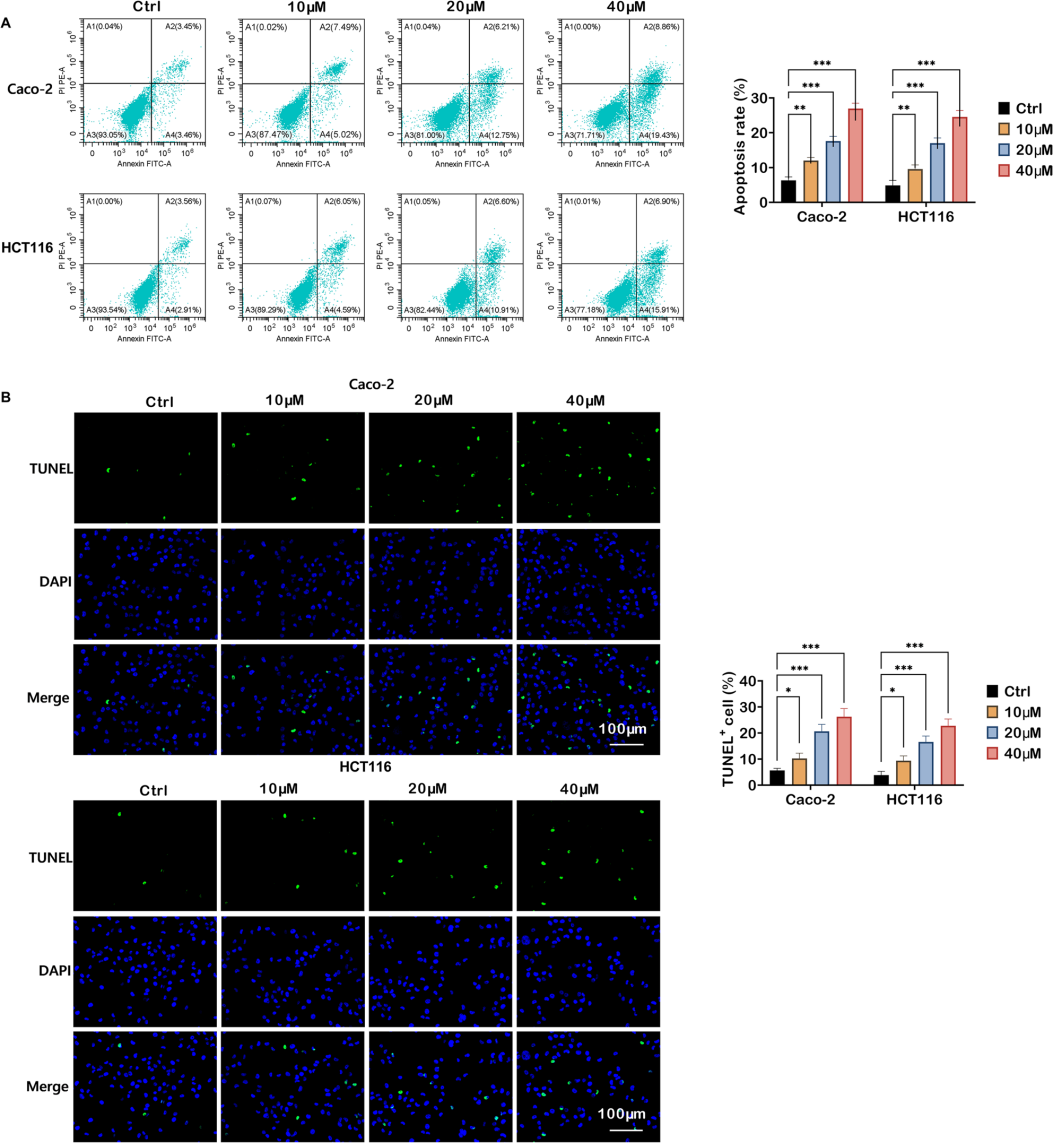
TRV130 induces apoptosis in colon cancer cells in a concentration-dependent manner. (**A**) Apoptosis of Caco-2 and HCT116 colon cancer cells treated with TRV130 (10, 20, and 40 µM) was analyzed by Annexin V-FITC/PI flow cytometry, and the apoptosis rate was quantified.(**B**) TUNEL staining was used to detect apoptotic cells in Caco-2 and HCT116 colon cancer cells after TRV130 treatment (10, 20, and 40 µM) (Scale bar = 100 μm). * *P* < 0.05, ** *P* < 0.01, *** *P* < 0.001

Subsequently, whether TRV130 elicited apoptosis in colon cancer cells was investigated. As depicted in Fig. 2A, flow cytometry revealed that TRV130 treatment (at 10, 20, and 40 µM) dose-dependently enhanced the apoptosis rate in both Caco-2 and HCT116 cells. Quantitative analysis further confirmed that TRV130 increased the percentage of apoptotic cells, with the most robust induction observed at 40 µM. To corroborate these findings, TUNEL staining was performed to visualize apoptotic cells (Fig. 2B). In the Ctrl group, only a few TUNEL-positive cells (green fluorescence) were observed in Caco-2 and HCT116 cells. However, upon TRV130 treatment, the count of TUNEL-positive cells was markedly elevated. In conclusion, these findings demonstrated that TRV130 triggered apoptotic processes in colon cancer cells.

### TRV130 represses migration and invasion of colon cancer cells

**Fig. 3:**
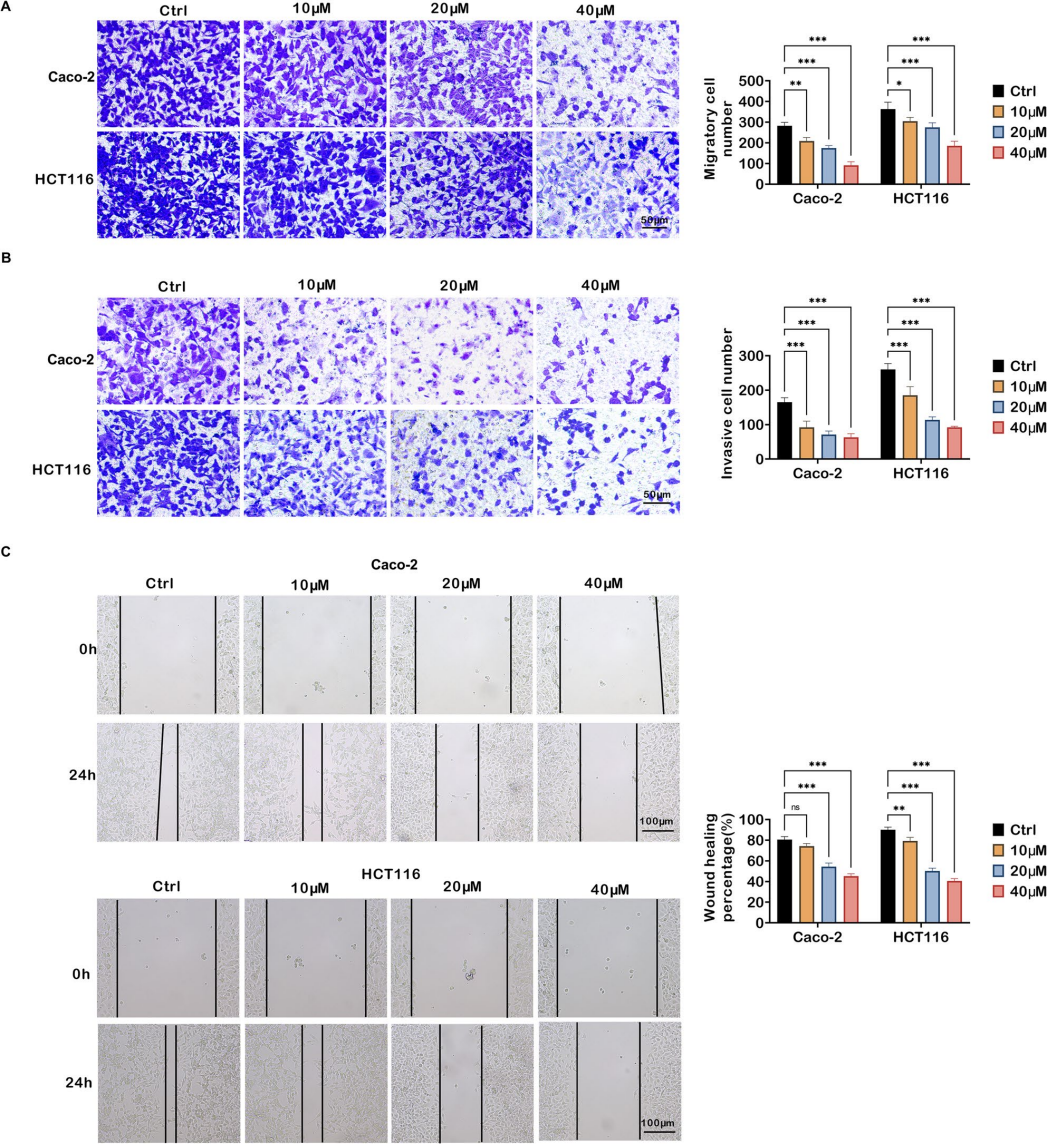
TRV130 suppresses migration and invasion of colon cancer cells. Caco-2 and HCT116 cells were treated with 10, 20, and 40 µM TRV130. (**A**-**B**) Transwell assays were performed to assess the migratory and invasive capacity of Caco-2 and HCT116 cells (Scale bar = 50 μm). (**C**) Wound healing assays were used to detect the migratory potential of Caco-2 and HCT116 cells; representative images at 0 h and 24 h were captured, and the wound closure percentage was quantified (Scale bar = 100 μm). * *P* < 0.05, ** *P* < 0.01, *** *P* < 0.001, ns, not significant

Transwell and wound healing assays were performed to assess the effects of TRV130 on the migration and invasion of colon cancer cell lines. Migration assay uncovered that TRV130 treatment reduced the number of migrated Caco-2 and HCT116 cells (Fig. 3A). A similar inhibitory trend was observed in invasion assay. TRV130 significantly decreased the number of invasive cells (Fig. 3B). Wound healing assay further illustrated the anti-migratory effect of TRV130 (Fig. 3C). After 24 h of incubation, the wound closure percentage in the Ctrl groups was notably higher than that in TRV130-treated groups. TRV130 at 20 µM and 40 µM dramatically reduced the wound closure rate in both Caco-2 and HCT116 cells, indicating a strong inhibition of cell migratory capacity. These data demonstrated that TRV130 constrained the migration and invasion of colon cancer cells.

### TRV130 regulates expression of cell proliferation- and apoptosis-associated proteins in colon cancer cells

**Fig. 4:**
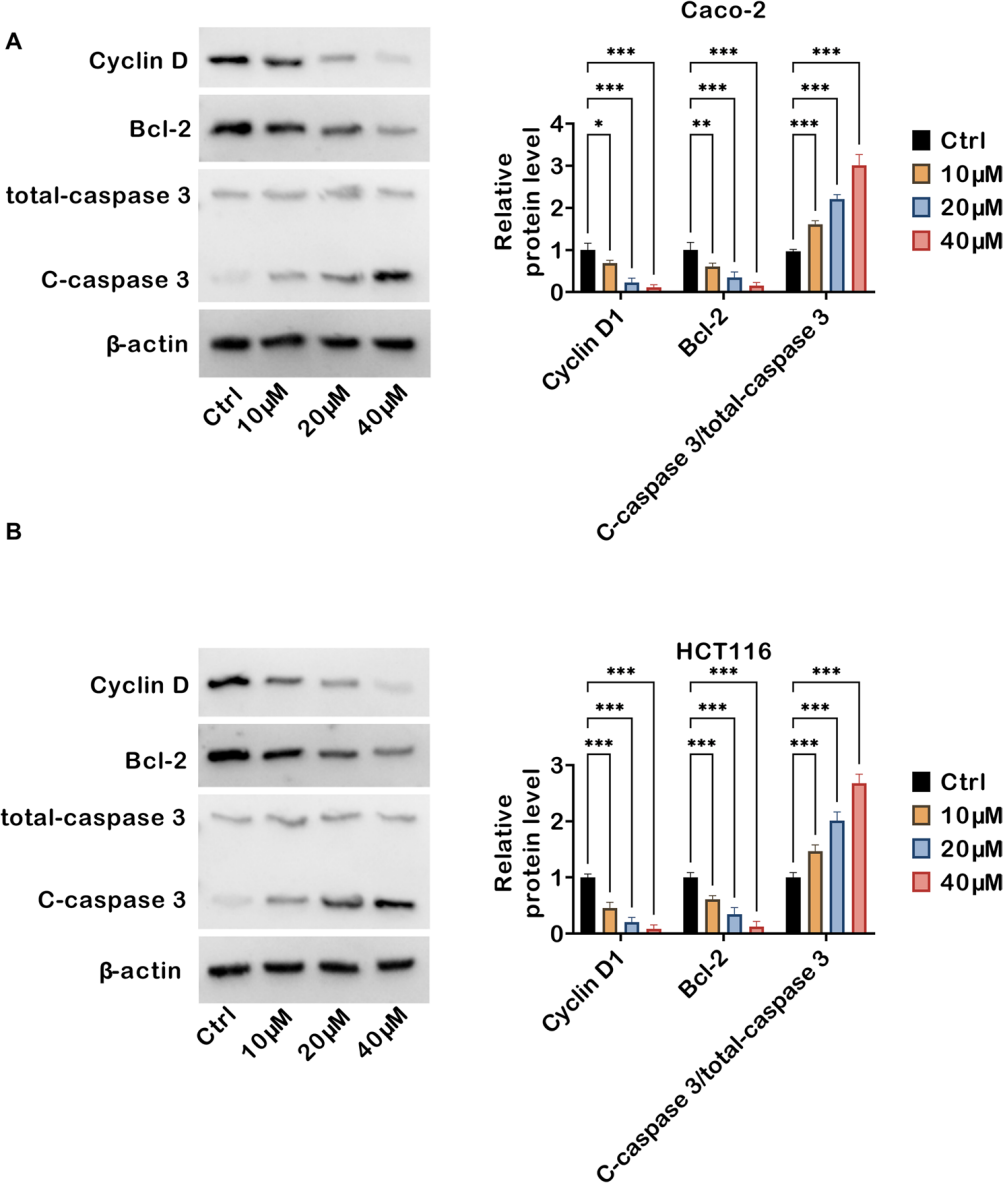
TRV130 modulates expression of cell proliferation- and apoptosis-related proteins in colon cancer cells. Caco-2 and HCT116 cells were treated with TRV130 (10, 20, and 40 µM). (**A**) Western blot analysis of Cyclin D1, Bcl-2, total-caspase 3, and C-caspase 3 expression levels in Caco-2 cells. (**B**) Western blot analysis of Cyclin D1, Bcl-2, total-caspase 3, and C-caspase 3 levels in HCT116 cells. * *P* < 0.05, ** *P* < 0.01, *** *P* < 0.001

The molecular mechanisms underlying TRV130-mediated cell proliferation suppression and apoptosis induction in colon cancer cells were further elucidated. In Caco-2 cells, TRV130 treatment (10, 20, and 40 µM) led to a concentration-dependent reduction in the protein levels of Cyclin D1 (a pivotal regulator promoting G1-to-S phase transition in the cell cycle) and Bcl-2 (an anti-apoptotic protein that suppresses caspase activation). Conversely, the cleaved form of caspase 3 (C-caspase 3, a hallmark of apoptosis execution) was markedly upregulated with increasing TRV130 concentration, while the total caspase 3 levels remained relatively stable (Fig. 4A). Consistently, in HCT116 cells, TRV130 also exerted a concentration-dependent inhibitory effect on Cyclin D1 and Bcl-2 expression, while simultaneously enhancing caspase 3 activation (evidenced by increased C-caspase 3 levels) (Fig. 4B). To further elaborate on the specific apoptotic pathways involved, markers of the intrinsic mitochondrial pathway (Bax, Bim) and extrinsic death receptor pathway (FasL) were detected: in both Caco-2 and HCT116 cells, TRV130 treatment dose-dependently upregulated the protein levels of these markers (Fig. S1), confirming that TRV130 induced apoptosis via simultaneous activation of both pathways. Taken together, these data indicated that TRV130 modulated colon cancer cell proliferation and apoptosis by regulating Cyclin D1 (cell cycle progression), Bcl-2 (apoptosis suppression), activating caspase 3 (apoptosis execution) expression, and engaging both intrinsic and extrinsic apoptotic pathways.

### TRV130 inhibits the hh signaling pathway in colon cancer cells

**Fig. 5:**
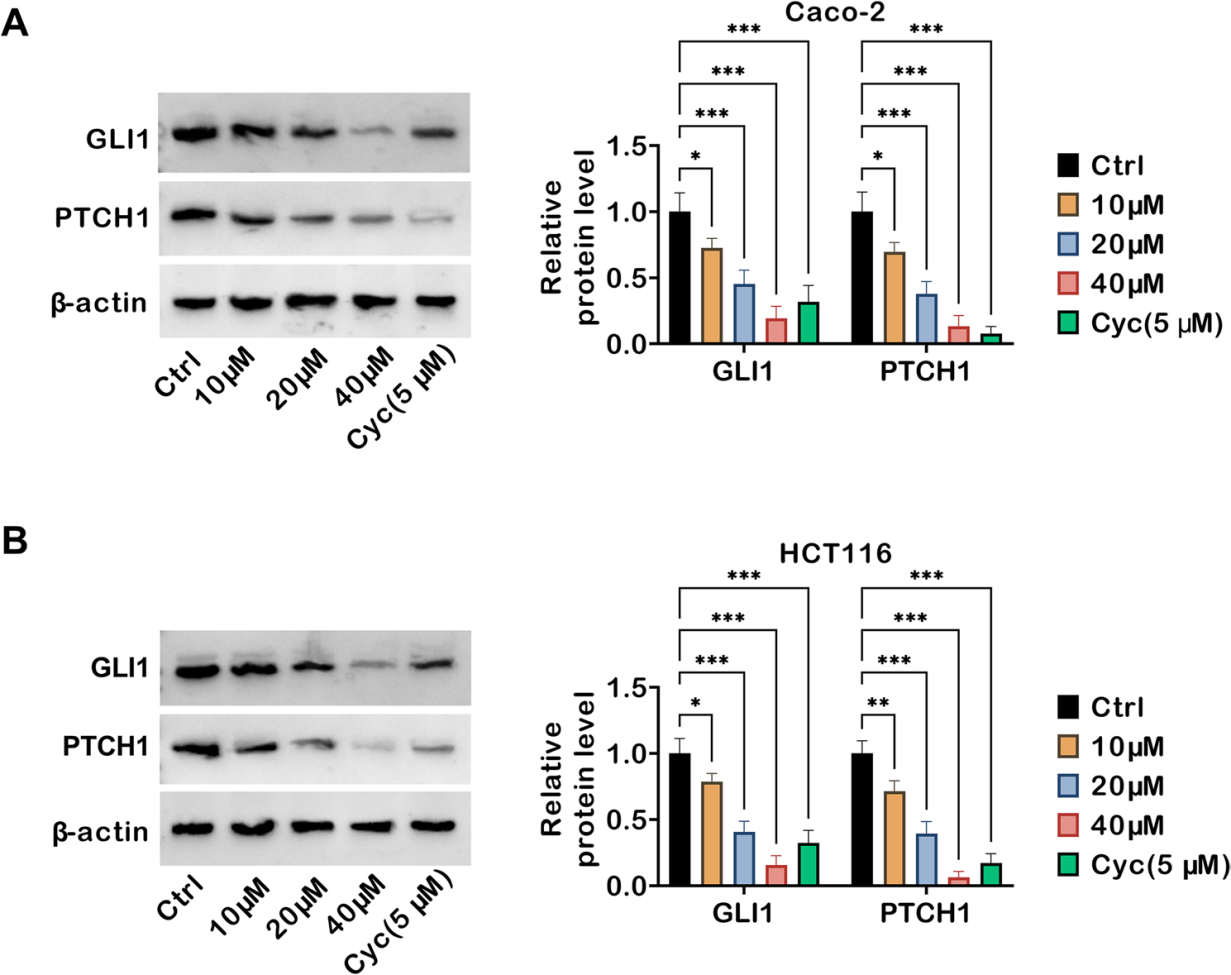
TRV130 inhibits the Hh signaling pathway in colon cancer cells. Caco-2 cells and HCT116 cells were treated TRV130 (10, 20, and 40 µM) or Cyc (5 µM) (**A**-**B**) Western blot analysis of GLI1 and PTCH1 (key Hh pathway proteins) expression levels in Caco-2 and HCT116 cells. * *P* < 0.05, ** *P* < 0.01, *** *P* < 0.001

To investigate the molecular mechanisms underlying TRV130’s anti-tumor effects, the key protein levels in the Hh signaling pathway, a crucial regulator of cancer cell growth and survival, were next examined. Western blot demonstrated that TRV130 treatment reduced the protein levels of GLI1 (a core transcriptional effector of the Hh pathway) and PTCH1 (an upstream receptor and pathway regulator) in Caco-2 cells. Notably, Cyc, a well-established Hh pathway inhibitor used as a positive control, also markedly decreased GLI1 and PTCH1 expression (Fig. 5A). Similarly, in HCT116 cells, TRV130 exerted a concentration-dependent inhibitory effect on GLI1 and PTCH1 expression. Cyc also suppressed Hh signaling as expected (Fig. 5B). Together, TRV130 inhibited the Hh signaling pathway in colon cancer cells by downregulating GLI1 and PTCH1, an effect that might contribute to its anti-tumor activity.

### TRV130 constrains tumor growth and modulates key pathway proteins in xenograft mice

**Fig. 6:**
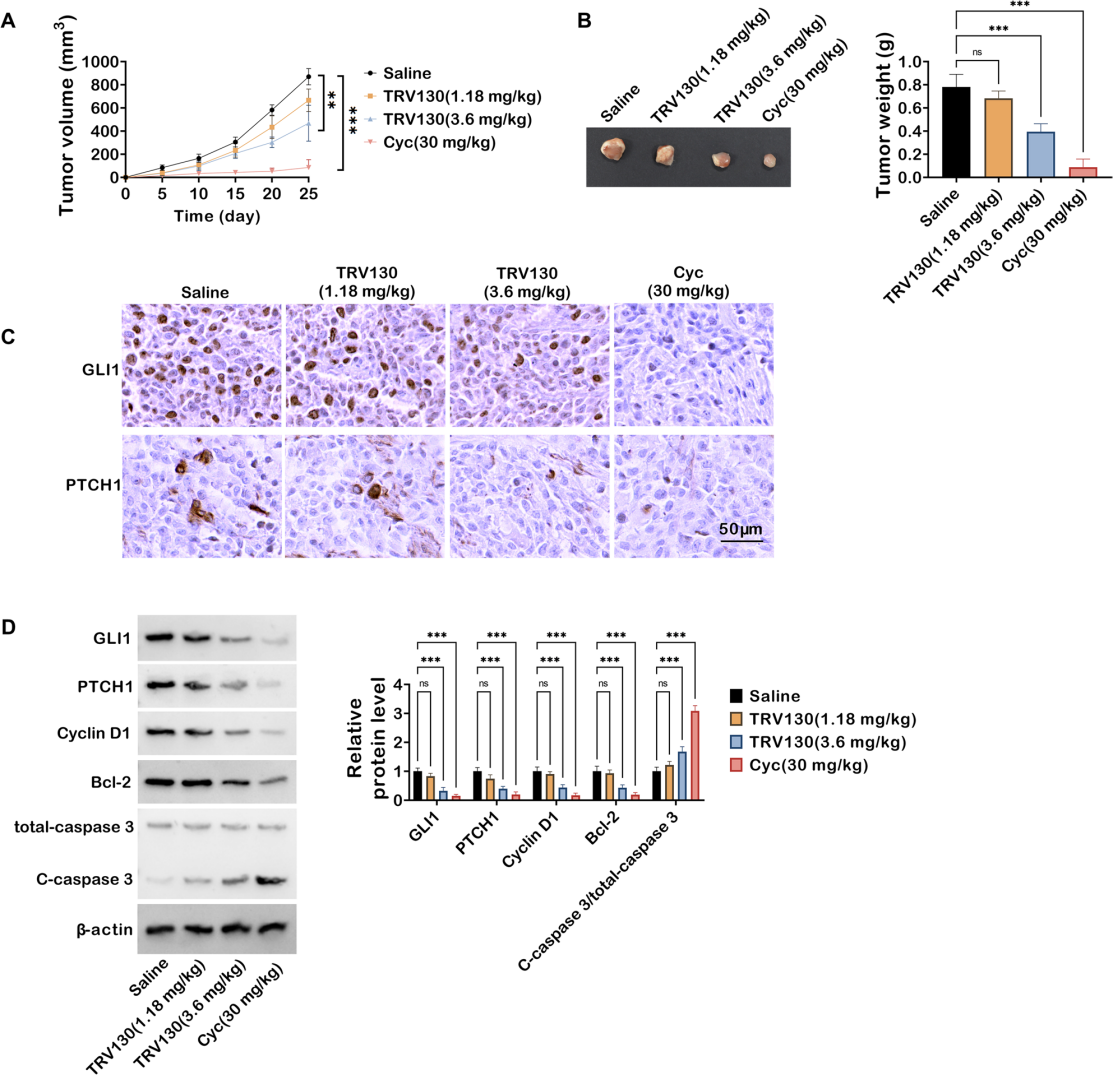
TRV130 constrains tumor growth and regulates Hh pathway and apoptosis-related proteins in HCT116 xenograft mice. Mice were divided into 4 groups: Saline, TRV130 (1.18 mg/kg), TRV130 (3.6 mg/kg), and Cyc (30 mg/kg). (**A**) Tumor volume was measured every 5 days in xenograft mice.(**B**) Representative images of excised tumors (left) and quantitative analysis of tumor weight (right) at the endpoint. (**C**) IHC staining of GLI1 and PTCH1 in tumor tissues; scale bar = 50 μm. (**D**) Western blot analysis of GLI1, PTCH1, Cyclin D1, Bcl-2, total-caspase 3, and C-caspase 3 in tumor tissues. ** *P* < 0.01, *** *P* < 0.001, ns, not significant

Building upon the in vitro findings that TRV130 suppressed colon cancer cell behaviors via the Hh pathway, its anti-tumor efficacy was next evaluated using a xenograft mouse model. Tumor volume was dynamically monitored over 25 days: mice in the saline control group exhibited rapid tumor growth, while TRV130-treated groups (1.18 mg/kg and 3.6 mg/kg) displayed dose-dependent inhibition of tumor expansion. Notably, Cyc also significantly suppressed tumor growth (Fig. 6A). Figure 6B presented representative images of excised tumors and quantitative analysis of tumor weight at the endpoint. Tumor weight in the TRV130 (3.6 mg/kg) group was markedly lower than that in the saline group, and Cyc also significantly reduced tumor weight, further confirming TRV130’s anti-tumor activity in vivo. Subsequently, IHC was performed to assess Hh pathway proteins in tumor tissues. Compared with the saline group, TRV130 treatment and Cyc notably decreased the positive staining of GLI1 and PTCH1, indicating suppression of the Hh pathway in vivo (Fig. 6C). Western blot analysis further validated these results at the protein level. TRV130 dose-dependently downregulated GLI1, PTCH1, Cyclin D1, and Bcl-2, while concurrently increasing the cleavage of caspase 3 (reflected by the elevated ratio of C-caspase 3 to total caspase 3). Cyc treatment showed consistent trends, reducing pro-proliferative and anti-apoptotic proteins and activating caspase 3 (Fig. 6D). In summary, these in vivo data demonstrated that TRV130 inhibited tumor growth in xenograft mice, at least in part by suppressing the Hh signaling pathway and modulating downstream cell proliferation and apoptosis-related proteins.

## Discussion

Colon cancer ranks among the highest in incidence and mortality worldwide, and its treatment still faces challenges such as a high metastasis rate and significant drug resistance [30, 31]. In recent years, novel therapeutic strategies targeting the regulation of tumor proliferation, apoptosis, and key signaling pathways have attracted considerable attention [32].

Uncontrolled proliferation, apoptosis resistance, and invasive/metastatic capabilities of tumor cells are the core biological basis for the progression and recurrence of colon cancer, as well as key targets in clinical treatment [33]. In vitro experiments in this work confirmed that TRV130 significantly inhibited proliferation of Caco-2 and HCT116 cells. This result aligns with the findings of previous studies on other tumors, in which certain agents inhibit tumor proliferation by interfering with cell cycle progression [34]. Furthermore, TRV130 significantly increased the apoptosis rate of colon cancer cells while suppressing their horizontal migration and basement membrane invasion capacities, suggesting that it might block the metastatic process by impairing the motility of tumor cells and their ability to degrade the extracellular matrix. Dysregulation of the cell cycle and abnormal apoptosis pathways are the core molecular mechanisms underlying uncontrolled proliferation in colon cancer, with Cyclin D1, Bcl-2, and members of the Caspase family serving as key regulatory factors [35–37]. Cyclin D1, known as the “switch molecule” for the G1-to-S phase transition, is overexpressed in colon cancer tissues and accelerates cell cycle progression [38, 39]. Bcl-2, as a classic anti-apoptotic protein, can block the initiation of apoptosis by inhibiting the opening of mitochondrial permeability transition pores (mPTP), and its expression is closely linked to chemoresistance and poor prognosis in patients with colon cancer [40, 41]. In this current work, TRV130 was found to downregulate Cyclin D1 and Bcl-2 protein expression in a concentration-dependent manner, while significantly activating Caspase 3. This molecular change closely aligns with its phenotypic effects of inhibiting proliferation and promoting apoptosis. This mechanism is consistent with that of CDK4/6 inhibitors (e.g., palbociclib), which exert their effects by inhibiting the Cyclin D-CDK4/6 complex [42, 43]. However, as a non-kinase inhibitor, the specificity and off-target effects of TRV130 require further investigation.

Aberrant activation of the Hh pathway can drive the expansion of cancer stem cells and the maintenance of malignant phenotypes by remodeling embryonic proliferation signals, and it has been confirmed to be strongly related to the occurrence, progression, chemoresistance, and metastasis of colon cancer [44, 45]. As an upstream negative regulator of the pathway, PTCH1 normally inhibits SMO; loss of PTCH1 function or downregulated PTCH1 expression relieves this inhibition, thereby activating the downstream transcription factor GLI1 [46, 47]. GLI1 can bind to the promoter regions of target genes, driving their expression and promoting tumor progression [48]. In this study, TRV130 downregulated the protein expression of PTCH1 and GLI1 in colon cancer cells, with effects consistent with those of the positive control drug Cyc. As the effector terminal of the pathway, the functional activation of GLI1 is a prerequisite for the metastasis of colon cancer. Following the downregulation of GLI1 by TRV130, Cyclin D1 and Bcl-2 levels were concomitantly decreased, and cleaved caspase 3 levels were increased, suggesting that the inhibitory effect of TRV130 on the Hh pathway could be translated into anti-tumor phenotypes through downstream effector molecules. The consistency between the anti-tumor effect of TRV130 and the downregulation of pathway molecules as well as the regulation of proliferation- and apoptosis-related proteins in in vivo experiments strongly suggests that Hh pathway inhibition is a core mechanism underlying the anti-colon cancer activity of TRV130.

Currently, clinically available Hh pathway inhibitors (e.g., vismodegib) can inhibit tumor growth, but their clinical application is limited by the high risk of acquired drug resistance (resulting from their targeting of SMO) and severe adverse reactions such as skin toxicity and dysgeusia [49, 50]. Although small-molecule drugs that directly target GLI1 can circumvent SMO-mediated resistance, their clinical translation is hindered by high off-target risks because the zinc finger structure of GLI1 shares high structural homology with that of other transcription factors, and no such drugs have entered Phase III clinical trials to date [51]. Notably, targeted regulation of distinct molecular pathways is effective for colon cancer treatment, as demonstrated by Gelsolin’s inhibition of colon cancer proliferation via the TNFR2/CASP10 death receptor pathway [52]. This supports TRV130’s value as a clinically approved analgesic with a unique Hh pathway-targeting mechanism, offering a novel translational alternative.

As a clinically approved biased µ-opioid receptor agonist for the treatment of moderate to severe acute pain, TRV130 has well-validated pharmacokinetic stability and clinical safety [53], which endows it with unique translational advantages in colon cancer treatment. Compared with de novo developed targeted drugs, the “known safety profile” of TRV130 can significantly shorten the preclinical toxicity evaluation cycle and reduce late-stage development risks. This holds important practical significance for colon cancer patients with concurrent cancer pain, as TRV130 is expected to address the dual needs of “pain management and tumor treatment” with a single agent, thereby reducing adverse reactions associated with polypharmacy.

Notably, previous studies have suggested that opioid drugs may influence tumor progression by regulating the tumor microenvironment [54], but this study did not investigate whether TRV130 mediates the inhibitory effect on the Hh pathway through the µ-opioid receptor. Future studies should clarify the association between the “TRV130-µ-opioid receptor-Hh pathway” components via receptor knockdown/knockout experiments to further refine the molecular mechanism network. Additionally, the subcutaneous tumor xenograft model used in this study did not simulate clinically common scenarios such as peritoneal, hepatic, or pulmonary metastasis of colon cancer, nor did it include clinical samples to verify correlation between Hh pathway molecule expression and TRV130 responsiveness. These limitations should be addressed in subsequent studies through orthotopic metastasis models and clinical specimen analysis.

In summary, TRV130 can inhibit the Hh signaling pathway, thereby suppressing colon cancer cell proliferation, inducing apoptosis, blocking invasion and metastasis, and effectively inhibiting tumor growth in nude mouse tumor xenograft models. This finding not only fills the gap in research on the anti-tumor effects of TRV130 but also addresses the target limitations of traditional Hh inhibitors, providing a novel “safe, controllable, and multi-effect synergistic” candidate drug for colon cancer treatment. In the future, combination therapy experiments using TRV130 with existing chemotherapeutic drugs can be further conducted to explore its potential in reversing drug resistance. Moreover, verifying its association with patient prognosis through clinical samples will lay the foundation for advancing its entry into clinical research for colon cancer.

## Supplementary Information

Supplementary Material 1.

Supplementary Material 2.

## Data Availability

Data sharing not applicable to this article as no datasets were generated or analysed during the current study.
