# Supplementary material for: TRV130 inhibits colon cancer progression via suppressing the Hedgehog signaling pathway: in vitro and in vivo evidence
Source: Hereditas. 2026 Jan 19;163:26. doi: 10.1186/s41065-026-00633-6 (PMC12911246; doi:10.1186/s41065-026-00633-6)

MagicMark XP Western蛋白分子量标准品

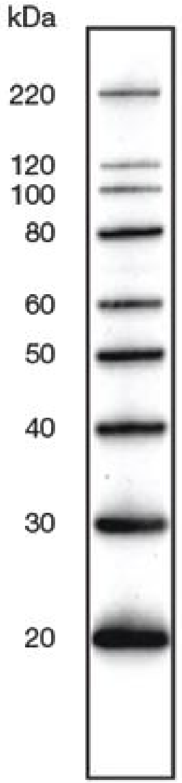

Fig 4 A

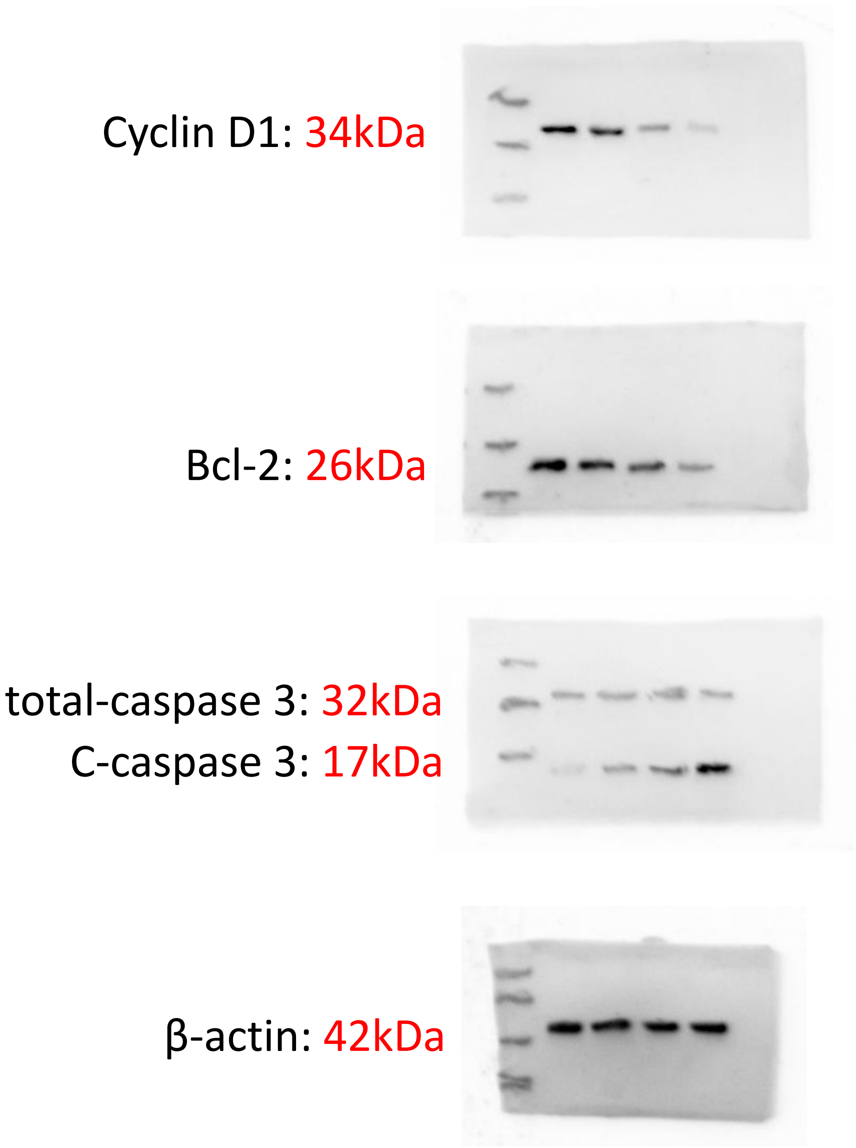

Cyclin D1: 34kDa
Bcl-2: 26kDa
total-caspase 3: 32kDa
C-caspase 3: 17kDa
β-actin: 42kDa

Fig 4 B

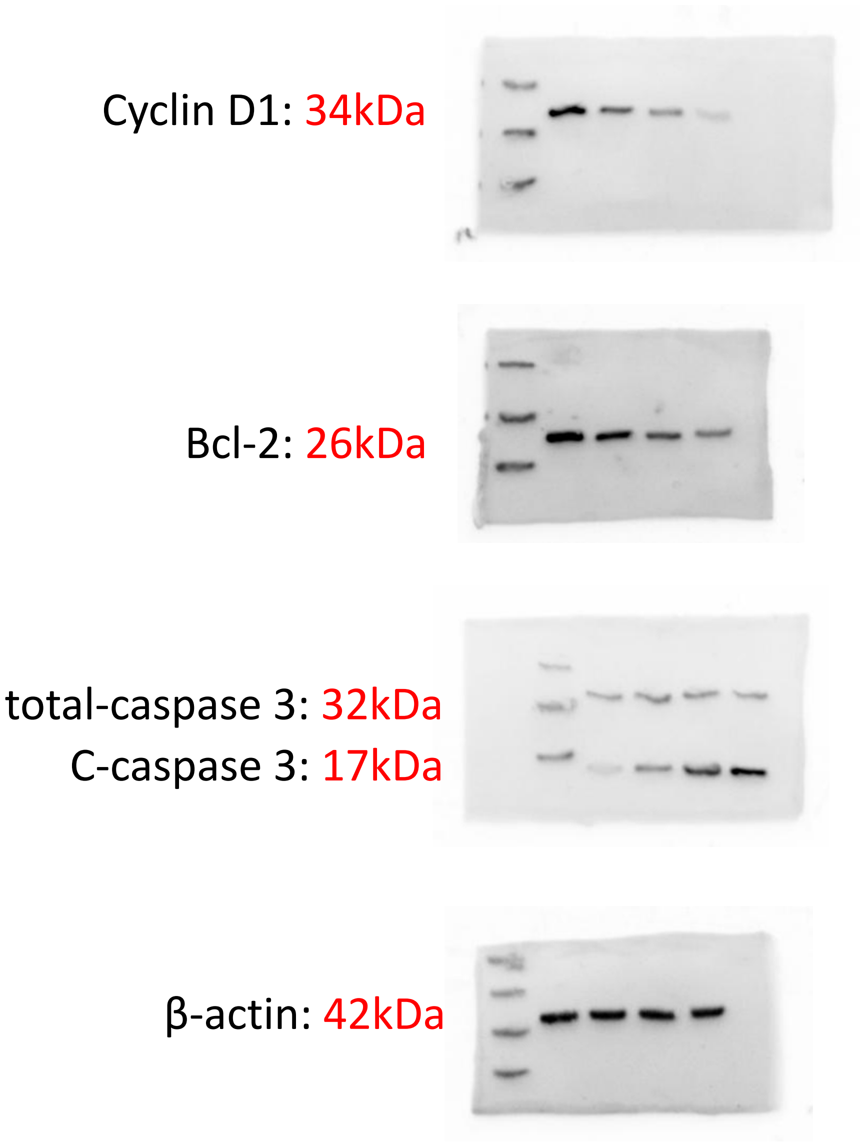

Cyclin D1: 34kDa
Bcl-2: 26kDa
total-caspase 3: 32kDa
C-caspase 3: 17kDa
β-actin: 42kDa

Fig 5 A

GLI1: 118 kDa

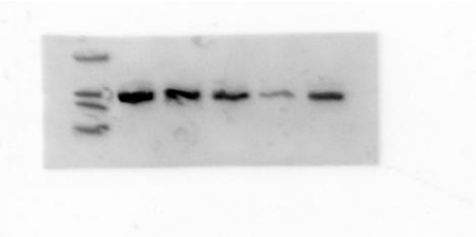

PTCH1: 161 kDa

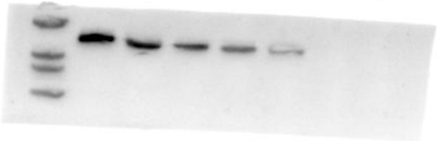

β-actin: 42kDa

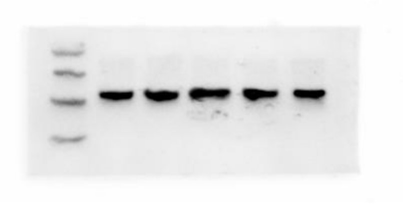

Fig 5 B

GLI1: 118 kDa

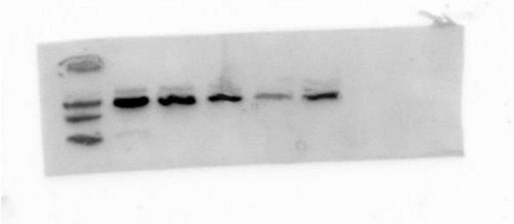

PTCH1: 161 kDa

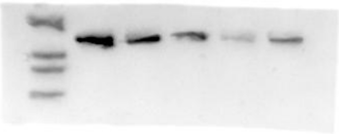

β-actin: 42kDa

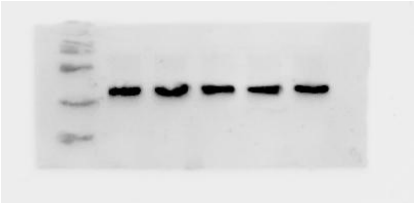

Fig 6D

GLI1: 118 kDa

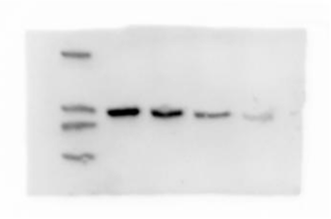

PTCH1: 161 kD

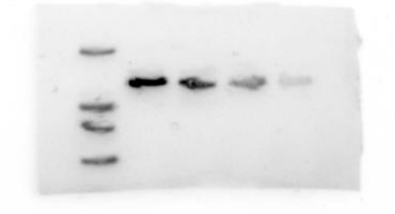

Cyclin D1: 34kDa

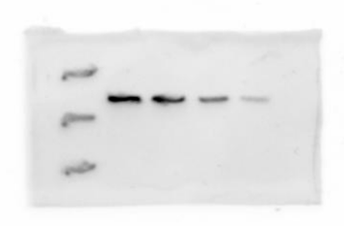

Bcl-2: 26kDa

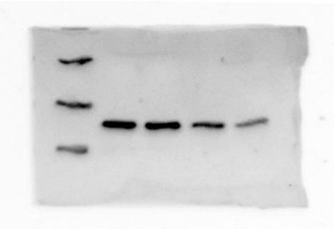

total-caspase 3: 32kDa

C-caspase 3: 17kDa

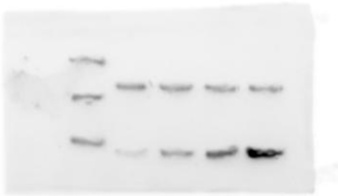

β-actin: 42kDa

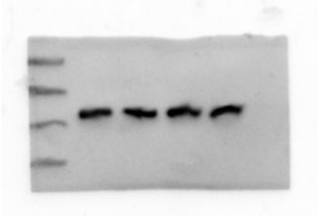

Supplement: Supplementary file 1 — Supplementary Material 1. [file 41065_2026_633_MOESM1_ESM.pdf]
