# Supplementary material for: TRV130 inhibits colon cancer progression via suppressing the Hedgehog signaling pathway: in vitro and in vivo evidence
Source: Hereditas. 2026 Jan 19;163:26. doi: 10.1186/s41065-026-00633-6 (PMC12911246; doi:10.1186/s41065-026-00633-6)
Supplement: Supplementary file 2 — Supplementary Material 2. [file 41065_2026_633_MOESM2_ESM.docx]

**TRV130 Inhibits Colon Cancer Progression via Suppressing the Hedgehog Signaling Pathway: *In Vitro* and *In Vivo* Evidence**

Yuanzhao Zhuang, Changcheng Jiang, Yuqing Guo, Jiaxiao Sun*

Department of Anesthesiology, Quanzhou first hospital Affiliated to Fujian Medical University, Quanzhou, Fujian Province, China.

***Corresponding author**: Jiaxiao Sun MD. Department of Anesthesiology, Quanzhou first hospital Affiliated to Fujian Medical University, No. 250, Dong Street, Licheng District 515041, Quanzhou, Fujian Province, China.

<TEL:+86-0595-22277597> Email: [sunjiaxiao87@163.com](mailto:sunjiaxiao87@163.com)

**Running title:** **TRV130’s Anti-Tumor Effect on Colon Cancer**

**Abstract**

**Background:** Colon cancer, characterized by high incidence and mortality, faces clinical challenges due to high recurrence rates and drug resistance. Aberrant activation of Hedgehog (Hh) signaling pathway is a key driver of colon cancer progression, making it a promising therapeutic target; however, current Hh inhibitors are limited by resistance and adverse effects. Drug repurposing offers a strategic alternative to accelerate oncology drug development. Oliceridine (TRV130) is a clinically approved, selective μ-opioid receptor agonist with a well-established safety profile for pain management. Notably, its potential anti-tumor activity and impact on oncogenic pathways like Hh signaling remain entirely unexplored. This study aims to investigate the anti-colon cancer efficacy of TRV130 and its underlying mechanisms, focusing on the Hh pathway, thereby evaluating its repurposing potential.

**Methods:** To establish the rationale for targeting the Hh pathway and the novelty of investigating TRV130, a focused literature review was conducted using PubMed and Web of Science databases (search period: 2000-2023), employing keywords including “colon cancer,” “Hedgehog pathway,” “drug repurposing,” and “TRV130/Oliceridine.” Based on this foundational evidence, the anti-tumor effects of TRV130 were systematically evaluated. Cell proliferation was assessed via MTT, EdU, and colony formation assays. Apoptosis was evaluated by flow cytometry and TUNEL assays. Migration and invasion were analyzed by Transwell and wound healing assays. The expressions of Cyclin D1, Bcl-2, Caspase 3, and Hh pathway proteins (PTCH1, GLI1) were detected by Western blot. A subcutaneous xenograft model was established in nude mice using HCT116 cells to validate *in vivo* efficacy, with tumor tissues analyzed by immunohistochemistry (IHC) and Western blot.

**Results:** TRV130 exhibited potent, dose-dependent inhibition of colon cancer cell proliferation, migration, and invasion. It induced apoptosis through both intrinsic and extrinsic pathways, as evidenced by the downregulation of Cyclin D1 and Bcl-2, and the upregulation of cleaved caspase 3. Mechanistically, TRV130 significantly suppressed the Hh signaling pathway, reducing the expression of its key effectors (GLI1 and PTCH1) to an extent comparable to cyclopamine (Cyc), a canonical Hh inhibitor. *In vivo*, TRV130 administration dose-dependently inhibited tumor growth in xenograft models, reducing both tumor volume and weight. IHC and Western blot analyses of tumor tissues confirmed the downregulation of Hh pathway proteins and pro-proliferative markers, alongside upregulation of apoptotic markers.

**Conclusion:** This study identifies TRV130 as a novel inhibitor of the Hh pathway, demonstrating its significant anti-colon cancer effects *in vitro* and *in vivo*. These findings reveal a previously unexplored, oncological mechanism for this clinically safe drug and support its repurposing as a promising therapeutic candidate, potentially offering an alternative to current Hh-targeted therapies. This work provides the foundational evidence for further development of TRV130 against colon cancer.

**Keywords:** Colon cancer, Oliceridine, Hedgehog, Proliferation, Apoptosis

**Introduction**

Colon cancer is a highly prevalent gastrointestinal malignancy worldwide, and its incidence continues to rise [^1^](#_ENREF_1). Most patients are diagnosed at advanced stages, accompanied by a risk of recurrence and distant metastasis [^2^](#_ENREF_2)^,^[^3^](#_ENREF_3). Currently, clinical regimens combining surgery with chemoradiotherapy and targeted therapy can prolong the survival of some patients; however, challenges including chemoresistance, postoperative recurrence, and severe adverse reactions still significantly limit improvements in prognosis [^4-6^](#_ENREF_4). Therefore, exploring novel therapeutic strategies with both efficacy and safety, and expanding the therapeutic potential of approved drugs, has become a key direction for breaking through the bottlenecks in colon cancer treatment.

In recent years, the development of natural product-derived extracts, plant-derived nanoparticles, and novel targeted formulations has also provided diverse insights for cancer therapy [^7-10^](#_ENREF_7). Concurrently, the strategy of drug repurposing, which identifies new therapeutic uses for approved drugs, has gained prominence for its potential to accelerate clinical translation by leveraging existing safety and pharmacokinetic data. In parallel, advances in green nanotechnology, such as the use of plant-derived silver nanoparticles as potent radiosensitizers or magnetic nanocomposites for targeted drug delivery, underscore the ongoing pursuit of safer, more sustainable anticancer platforms [^11-15^](#_ENREF_11). Currently, the use of small-molecule drugs targeting G protein-coupled receptors (GPCRs) is an important strategy for cancer treatment. Research on GPCR-targeted drugs has made considerable progress [^16^](#_ENREF_16)^,^[^17^](#_ENREF_17). For instance, targeting adrenergic receptor pathways has shown promise in modulating tumor biology in cancers such as glioma [^18^](#_ENREF_18), highlighting the broad therapeutic potential of modulating GPCR signaling in oncology. Oliceridine (TRV130) is a clinically approved biased μ-opioid receptor agonist (a class of GPCR-targeted small-molecules) used for treating moderate to severe acute pain. By selectively activating G protein signaling pathways and attenuating β-arrestin-mediated signal transduction, it retains potent analgesic effects while markedly reducing adverse reactions such as respiratory depression and gastrointestinal symptoms associated with traditional opioid drugs [^19^](#_ENREF_19)^,^[^20^](#_ENREF_20). To date, the clinical application of TRV130 has covered multiple settings including postoperative pain and traumatic pain, and its pharmacokinetic stability and safety are well-documented [^21^](#_ENREF_21). However, no studies have investigated whether TRV130 possesses anti-tumor activity, especially its effect on colon cancer and the associated molecular mechanisms. This represents a critical gap in exploring the expansion of its therapeutic utility.

To address this gap, it is critical to anchor the investigation in key signaling pathways that drive colon cancer progression, among which the Hedgehog (Hh) signaling pathway stands out as a well-validated core regulator of tumorigenesis and malignancy. It is primarily quiescent in normal adult tissues but is dysregulated and activated in a range of malignancies including colon cancer, with its activation level tightly linked to tumor malignancy grade and patient prognosis [^22^](#_ENREF_22)^,^[^23^](#_ENREF_23). Notably, while other oncogenic factors (e.g., AEG-1) and posttranscriptional events (e.g., alternative polyadenylation) contribute to colon cancer progression, the Hh pathway remains a well-validated core driver, making it a priority target for therapeutic intervention. The Hh pathway is activated when the transmembrane receptor patched 1 (PTCH1) relieves its inhibition of Smoothened (SMO); activated SMO then drives the transcription factor GLI family zinc finger 1 (GLI1), which in turn promotes proliferation, suppresses apoptosis, and enhances invasiveness [^24-27^](#_ENREF_24). Although studies have confirmed that targeting the Hh pathway can significantly attenuate the malignant phenotype of colon cancer, current clinically available pathway inhibitors still have issues such as high toxicity and susceptibility to acquired drug resistance [^28^](#_ENREF_28). Moreover, recent comprehensive analyses of colon adenocarcinoma have revealed that posttranscriptional regulatory events, such as alternative polyadenylation (APA), also contribute to tumor progression [^29^](#_ENREF_29). Specifically, dynamic APA changes in genes involved in cell-cycle regulation and extracellular matrix remodeling have been observed during tumorigenesis, which may alter gene expression patterns to promote cancer cell malignant transformation. These findings underscore the multi-layered complexity of colon cancer pathogenesis, further supporting the need for novel therapeutic agents that can target key signaling nodes with an improved safety and efficacy profile.

Based on this, this study systematically investigates the anti-colon cancer activity of TRV130, with a particular focus on clarifying its regulatory relationship with the Hh signaling pathway and the core underlying molecular mechanisms. This work aims to fill the research gap regarding the anti-tumor effect of TRV130, verify its clinical applicability for colon cancer treatment, and meanwhile provide a novel, safe, and controllable candidate drug for Hh pathway-targeted therapy, thereby laying an experimental foundation and providing theoretical support for breaking through the bottlenecks in colon cancer treatment.

**Materials and methods**

**Cell lines**

The human colon cancer cell lines Caco-2 and HCT116 were obtained from Wuhan Procell (Wuhan, China). HCT116 cells harbor a KRAS mutation (G13D) and wild-type p53, with low basal activity of the Hh signaling pathway but detectable expression of key signaling components (e.g., SMO, GLI1). Caco-2 cells are KRAS wild-type but carry a p53 mutation (R273H), exhibiting moderate baseline activation of the Hh pathway—likely linked to their differentiation status. Caco-2 cells were cultured in MEM containing non-essential amino acids (NEAA) (Procell, Wuhan, China, Cat. no. PM150410) supplemented with 20% fetal bovine serum (FBS) (Procell, Cat. no. 164210) and 1% penicillin-streptomycin (P/S) (Procell, Cat. no. PB180120). HCT116 cells were maintained in McCoy’s 5A medium (Procell, Cat. no. PM150710) containing 10% FBS (Procell, Cat. no. 164210) and 1% P/S (Procell, Cat. no. PB180120). Both cell lines were incubated at 37℃ in a humidified atmosphere of 95% air and 5% CO_2_. Cells were treated with TRV130 (InvivoChem, Libertyville, IL, USA, Cat. no. V50861) at different concentrations (2.5, 5, 10, 20, 40, 80, and 160 μM) for 48 hours in preliminary dose-response experiments. For subsequent experiments, cells were divided into four groups: control (Ctrl), 10 μM TRV130, 20 μM TRV130, and 40 μM TRV130. For subsequent experiments, cells were divided into four groups: Ctrl, 10 μM TRV130, 20 μM TRV130, and 40 μM TRV130. For experiments specifically focused on investigating the Hh signaling pathway, Caco-2 and HCT116 cells were also treated with 5 μM cyclopamine (Cyc; MedChemExpress, Monmouth Junction, NJ, USA), a positive control for Hh pathway inhibition.

**Cell viability assay**

MTT Cell Proliferation and Cytotoxicity Assay Kit (Beyotime, Shanghai, China, Cat. no. C0009S) was employed to assess cell viability. 100 μL of cell suspension (1000 cells) was seeded into a 96-well plate for overnight incubation. After that, 10 μL of MTT solution was introduced, and the mixture was then incubated for an additional 4 hours. Subsequently, 100 μL of formazan solubilization solution was added, and the incubation was continued until the formazan crystals were dissolved. Finally, the absorbance was measured at 570 nm.

**Cell proliferation detection**

Cell proliferation was assessed using the BeyoClick™ EdU Cell Proliferation Kit with AF488 (Beyotime, Cat. no. C0071S). Prior to the experiment, the 2× EdU working solution was pre-warmed to 37℃ and added to wells at a 1:1 ratio with the existing culture medium. Following completion of EdU labeling, cells were sequentially fixed and permeabilized. The Click reaction solution was introduced, followed by incubation of the cells under dark conditions for 30 minutes. Nuclei were stained with 4’,6-Diamidino-2-Phenylindole, dihydrochloride (DAPI) (Beyotime), and EdU^+^ cells were counted to calculate the proliferation rate.

**Colony formation assay**

Cells were seeded in 6-well plates (500 cells per well) and incubated at 37℃ for 14 days. When visible colonies formed, the medium was carefully removed, and cells were fixed with 4% paraformaldehyde (Sigma-Aldrich, St. Louis, MO, USA), followed by staining with 0.1% crystal violet solution (Beyotime). After the cells were thoroughly washed with phosphate-buffered saline (PBS), the plates were air-dried, and colonies were counted under a light microscope to quantify clonogenic potential.

**Flow cytometry**

Apoptosis was evaluated using the Annexin V-FITC/PI Apoptosis Detection Kit (Yeasen, Shanghai, China, Cat. no. 40302ES50). Cells were digested and collected. The harvested cells were washed twice with pre-cooled PBS, yielding 5×10^5^ cells per sample. After removing PBS, cells were resuspended in 100 μL of 1× Binding Buffer, after which 5 μL Annexin V-FITC and 10 μL PI were added sequentially. The mixture was incubated for 15 minutes in the dark. Then, 400 μL of 1× Binding Buffer was added, and samples were analyzed using a flow cytometer within 1 hour.

**Cell apoptosis assessment**

Cell apoptosis was further evaluated using the CoraLite®594 TUNEL Apoptosis Detection Kit (Proteintech, Wuhan, China; Cat. no. PF00006). Cells were washed twice with PBS, followed by fixation and permeabilization. TUNEL reaction solution was prepared in strict accordance with the kit manufacturer’s protocols, with each sample receiving 50 μL of reaction solution containing 2 μL TdT enzyme. Cells were incubated at 37℃ for 60 minutes in the dark, after which the reaction solution was removed and the cells were rinsed with 1×PBS. Subsequently, cells were washed three times with a buffer consisting of 0.1% Triton X-100 and 5 mg/mL bovine serum albumin (BSA) prepared in PBS. Each sample was then treated with DAPI staining solution (2 μg/mL) for 10 minutes. Following staining, the samples were washed before analysis under a fluorescence microscope.

**Transwell assay**

2×10^4^ cells suspended in serum-free medium were added to the upper chambers of 24-well Transwell inserts (8-μm pore size) (Corning, Tewksbury, MA, USA). For invasion detection, the upper chambers were pre-coated with Matrigel (1:8 dilution in serum-free medium) (Corning, Bedford, MA, USA) and incubated at 37℃ for 30 minutes to form a gel. The lower chambers were loaded with complete medium containing 10% FBS. Following a 24-hour incubation at 37℃, remaining non-migrated (or non-invaded) cells on the upper surface of the membrane were softly wiped off. Cells on the lower surface were fixed with 4% paraformaldehyde (Beyotime) for 15 minutes and stained with 0.1% crystal violet (Beyotime) for 20 minutes. Five random fields per insert were imaged under a light microscope, and cells were counted to quantify migration and invasion capacity.

**Wound healing assay**

Cells were seeded in 6-well plates and cultured at 37℃ until reaching 90% confluence. A straight, consistent scratch was introduced into the cell monolayer via a sterile 200 μL pipette tip. Subsequent rinsing of the monolayer with serum-free medium (twice) eliminated detached cells and debris, after which the residual cells were incubated in fresh medium. Images of the wound area were captured at 0 h and 24 h using an inverted microscope. The wound width was measured at three random positions per image using ImageJ software, and the migration rate was calculated to quantify cell migration capacity.

**Western blot**

Proteins were extracted from cells using RIPA Lysis and Extraction Buffer (Thermo Fisher Scientific, Waltham, MA, USA), and protein concentrations were determined by BCA assay. After electrophoresis, separated proteins were transferred to PVDF membranes (Millipore, Billerica, MA, USA), which were then blocked with 5% non-fat milk for 1 hour at room temperature. Membranes were incubated with primary antibodies against Cyclin D1 (1:10000, Proteintech, Cat. no. 26939-1-AP), Bcl-2 (1:10000, Proteintech, Cat. no. 80313-1-RR), caspase 3 (1:800, Proteintech, Cat. no. 19677-1-AP), Cleaved-Caspase 3 (C-caspase 3; 1:1000, Proteintech, Cat. no. 25128-1-AP), β-actin (1:10000, Proteintech, Cat. no. 66009-1-Ig), GLI1 (1:20000, Proteintech, Cat. no. 66905-1-Ig), PTCH1 (1:1000, Cell Signaling Technology, Danvers, MA, USA, Cat. no. 2468S), Bax (1:2000, Proteintech, Cat. no. 60267-1-Ig), Bim (1:500, Proteintech, Cat. no. 22037-1-AP), and FasL (1:1000, Cell Signaling Technology, Cat. no. 68405) overnight at 4℃, followed by HRP-conjugated anti-rabbit/mouse secondary antibodies (1:1000, Proteintech, Cat. no. SA00001-2/RGAM001) for 1 hour at room temperature. After washing, protein bands were visualized using Clarity^TM^ Western ECL Substrate Kit (Bio-Rad, Shanghai, China) and a chemiluminescence imaging system. Band intensities were quantified using ImageJ software and normalized to internal controls.

**Xenograft model**

Female BALB/c nude mice (4-6 weeks old) were purchased from Beijing SPF Biotechnology Co., Ltd. (Beijing, China). For the establishment of subcutaneous colon cancer xenografts, HCT116 cells (5×10^6^ cells in 100 μL PBS) were subcutaneously injected into the right dorsal flank of each nude mouse. 14 days after cell inoculation, the *in vivo* TRV130 doses (1.18 mg/kg and 3.6 mg/kg) were selected based on PK/PD integration analysis (1.18 mg/kg as the minimum effective dose, 3.6 mg/kg achieving clinically equivalent exposure) and dose-escalation pilot experiments (no severe toxicity observed), with their converted human equivalent doses overlapping the clinical Phase I range to ensure clinical relevance. Mice were randomly divided into 4 groups (n=5/group): control group (intraperitoneal injection of normal saline), TRV130 low-dose group (L, 1.18 mg/kg, intraperitoneal injection), TRV130 high-dose group (H, 3.6 mg/kg, intraperitoneal injection), and Cyc group (30 mg/kg, intraperitoneal injection). Administration was performed once daily for 25 consecutive days. Mouse body weight and tumor volume (V = 0.5 × length × width^2^, measured with a vernier caliper) were recorded every 5 days. Upon completion of the experiment, mice were euthanized, and tumors were dissected and weighed to evaluate the anti-tumor efficacy of TRV130. All animal procedures were approved by the Institutional Animal Care and Use Committee (IACUC) of Quanzhou first hospital Affiliated to Fujian Medical University and strictly followed the 3R principles of animal welfare.

**Immunohistochemistry (IHC)**

IHC was performed on paraffin-embedded tumor tissue sections (4-μm thick). Sections were deparaffinized in xylene and rehydrated through a graded ethanol series, after which antigen retrieval was conducted in citrate buffer (pH 6.0) with microwave heating. Endogenous peroxidase activity was blocked with 3% H_2_O_2_ in methanol for 10 minutes, and nonspecific binding was inhibited by incubating sections with 5% BSA at room temperature for 1 hour. Sections were then incubated with anti-GLI1 (Proteintech, Cat. no. 66905-1-Ig) and anti-PTCH1 (Cell Signaling Technology, Cat. no. 2468S) overnight at 4℃, followed by HRP-conjugated secondary antibodies (Proteintech, Cat. no. SA00001-2/RGAM001) for 1 hour at room temperature. After washing with PBS, immunoreactivity was visualized using 3,3’-diaminobenzidine (DAB) substrate, and sections were counterstained with hematoxylin to label nuclei. Finally, sections were dehydrated, cleared in xylene, mounted with neutral mounting medium, and observed under a light microscope.

**Statistical analysis**

Statistical analyses were performed using GraphPad Prism 9.0 software (GraphPad Software, Inc., USA), and data were presented as mean ± standard deviation (SD). One-way analysis of variance (one-way ANOVA) was applied to compare differences among multiple groups with a single independent variable. Two-way ANOVA was used to analyze data involving two independent variables and their interaction. A *P* value < 0.05 was considered statistically significant.

**Results**

**TRV130 suppresses colon cancer cell proliferation**

To explore the impacts of TRV130 on the proliferative activity of colon cancer cells, a series of experiments were performed in Caco-2 and HCT116 cells. As shown in Fig. 1A, after 24-hour treatment with TRV130 at varying concentrations (2.5, 5, 10, 20, 40, 80, and 160 μM), the viability of colon cancer cells was significantly decreased in a concentration-dependent manner. To further assess cell proliferation, EdU incorporation assays were conducted. Relative to the Ctrl group, the proportion of EdU-positive cells in Caco-2 and HCT116 cells remarkably decreased as TRV130 concentrations increased (Fig. 1B). Colony formation assay revealed that TRV130 treatment reduced the number of colonies formed by Caco-2 and HCT116 cells (Fig. 1C). Collectively, TRV130 exerted a suppressive effect on colon cancer cell proliferation.

**TRV130 induces apoptosis in colon cancer cells**

Subsequently, whether TRV130 elicited apoptosis in colon cancer cells was investigated. As depicted in Fig. 2A, flow cytometry revealed that TRV130 treatment (at 10, 20, and 40 μM) dose-dependently enhanced the apoptosis rate in both Caco-2 and HCT116 cells. Quantitative analysis further confirmed that TRV130 increased the percentage of apoptotic cells, with the most robust induction observed at 40 μM. To corroborate these findings, TUNEL staining was performed to visualize apoptotic cells (Fig. 2B). In the Ctrl group, only a few TUNEL-positive cells (green fluorescence) were observed in Caco-2 and HCT116 cells. However, upon TRV130 treatment, the count of TUNEL-positive cells was markedly elevated. In conclusion, these findings demonstrated that TRV130 triggered apoptotic processes in colon cancer cells.

**TRV130 represses migration and invasion of colon cancer cells**

Transwell and wound healing assays were performed to assess the effects of TRV130 on the migration and invasion of colon cancer cell lines. Migration assay uncovered that TRV130 treatment reduced the number of migrated Caco-2 and HCT116 cells (Fig. 3A). A similar inhibitory trend was observed in invasion assay. TRV130 significantly decreased the number of invasive cells (Fig. 3B). Wound healing assay further illustrated the anti-migratory effect of TRV130 (Fig. 3C). After 24 hours of incubation, the wound closure percentage in the Ctrl groups was notably higher than that in TRV130-treated groups. TRV130 at 20 μM and 40 μM dramatically reduced the wound closure rate in both Caco-2 and HCT116 cells, indicating a strong inhibition of cell migratory capacity. These data demonstrated that TRV130 constrained the migration and invasion of colon cancer cells.

**TRV130 regulates expression of cell proliferation- and apoptosis-associated proteins in colon cancer cells**

The molecular mechanisms underlying TRV130-mediated cell proliferation suppression and apoptosis induction in colon cancer cells were further elucidated. In Caco-2 cells, TRV130 treatment (10, 20, and 40 μM) led to a concentration-dependent reduction in the protein levels of Cyclin D1 (a pivotal regulator promoting G1-to-S phase transition in the cell cycle) and Bcl-2 (an anti-apoptotic protein that suppresses caspase activation). Conversely, the cleaved form of caspase 3 (C-caspase 3, a hallmark of apoptosis execution) was markedly upregulated with increasing TRV130 concentration, while the total caspase 3 levels remained relatively stable (Fig. 4A). Consistently, in HCT116 cells, TRV130 also exerted a concentration-dependent inhibitory effect on Cyclin D1 and Bcl-2 expression, while simultaneously enhancing caspase 3 activation (evidenced by increased C-caspase 3 levels) (Fig. 4B). To further elaborate on the specific apoptotic pathways involved, markers of the intrinsic mitochondrial pathway (Bax, Bim) and extrinsic death receptor pathway (FasL) were detected: in both Caco-2 and HCT116 cells, TRV130 treatment dose-dependently upregulated the protein levels of these markers (Fig. S1), confirming that TRV130 induced apoptosis via simultaneous activation of both pathways. Taken together, these data indicated that TRV130 modulated colon cancer cell proliferation and apoptosis by regulating Cyclin D1 (cell cycle progression), Bcl-2 (apoptosis suppression), activating caspase 3 (apoptosis execution) expression, and engaging both intrinsic and extrinsic apoptotic pathways.

**TRV130 inhibits the Hh signaling pathway in colon cancer cells**

To investigate the molecular mechanisms underlying TRV130’s anti-tumor effects, the key protein levels in the Hh signaling pathway, a crucial regulator of cancer cell growth and survival, were next examined. Western blot demonstrated that TRV130 treatment reduced the protein levels of GLI1 (a core transcriptional effector of the Hh pathway) and PTCH1 (an upstream receptor and pathway regulator) in Caco-2 cells. Notably, Cyc, a well-established Hh pathway inhibitor used as a positive control, also markedly decreased GLI1 and PTCH1 expression (Fig. 5A). Similarly, in HCT116 cells, TRV130 exerted a concentration-dependent inhibitory effect on GLI1 and PTCH1 expression. Cyc also suppressed Hh signaling as expected (Fig. 5B). Together, TRV130 inhibited the Hh signaling pathway in colon cancer cells by downregulating GLI1 and PTCH1, an effect that might contribute to its anti-tumor activity.

**TRV130 constrains tumor growth and modulates key pathway proteins in xenograft mice**

Building upon the *in vitro* findings that TRV130 suppressed colon cancer cell behaviors via the Hh pathway, its anti-tumor efficacy was next evaluated using a xenograft mouse model. Tumor volume was dynamically monitored over 25 days: mice in the saline control group exhibited rapid tumor growth, while TRV130-treated groups (1.18 mg/kg and 3.6 mg/kg) displayed dose-dependent inhibition of tumor expansion. Notably, Cyc also significantly suppressed tumor growth (Fig. 6A). Fig. 6B presented representative images of excised tumors and quantitative analysis of tumor weight at the endpoint. Tumor weight in the TRV130 (3.6 mg/kg) group was markedly lower than that in the saline group, and Cyc also significantly reduced tumor weight, further confirming TRV130’s anti-tumor activity *in vivo*. Subsequently, IHC was performed to assess Hh pathway proteins in tumor tissues. Compared with the saline group, TRV130 treatment and Cyc notably decreased the positive staining of GLI1 and PTCH1, indicating suppression of the Hh pathway *in vivo* (Fig. 6C). Western blot analysis further validated these results at the protein level. TRV130 dose-dependently downregulated GLI1, PTCH1, Cyclin D1, and Bcl-2, while concurrently increasing the cleavage of caspase 3 (reflected by the elevated ratio of C-caspase 3 to total caspase 3). Cyc treatment showed consistent trends, reducing pro-proliferative and anti-apoptotic proteins and activating caspase 3 (Fig. 6D). In summary, these *in vivo* data demonstrated that TRV130 inhibited tumor growth in xenograft mice, at least in part by suppressing the Hh signaling pathway and modulating downstream cell proliferation and apoptosis-related proteins.

**Discussion**

Colon cancer ranks among the highest in incidence and mortality worldwide, and its treatment still faces challenges such as a high metastasis rate and significant drug resistance [^30^](#_ENREF_30)^,^[^31^](#_ENREF_31). In recent years, novel therapeutic strategies targeting the regulation of tumor proliferation, apoptosis, and key signaling pathways have attracted considerable attention [^32^](#_ENREF_32).

Uncontrolled proliferation, apoptosis resistance, and invasive/metastatic capabilities of tumor cells are the core biological basis for the progression and recurrence of colon cancer, as well as key targets in clinical treatment [^33^](#_ENREF_33). *In vitro* experiments in this work confirmed that TRV130 significantly inhibited proliferation of Caco-2 and HCT116 cells. This result aligns with the findings of previous studies on other tumors, in which certain agents inhibit tumor proliferation by interfering with cell cycle progression [^34^](#_ENREF_34). Furthermore, TRV130 significantly increased the apoptosis rate of colon cancer cells while suppressing their horizontal migration and basement membrane invasion capacities, suggesting that it might block the metastatic process by impairing the motility of tumor cells and their ability to degrade the extracellular matrix. Dysregulation of the cell cycle and abnormal apoptosis pathways are the core molecular mechanisms underlying uncontrolled proliferation in colon cancer, with Cyclin D1, Bcl-2, and members of the Caspase family serving as key regulatory factors [^35-37^](#_ENREF_35). Cyclin D1, known as the “switch molecule” for the G1-to-S phase transition, is overexpressed in colon cancer tissues and accelerates cell cycle progression [^38^](#_ENREF_38)^,^[^39^](#_ENREF_39). Bcl-2, as a classic anti-apoptotic protein, can block the initiation of apoptosis by inhibiting the opening of mitochondrial permeability transition pores (mPTP), and its expression is closely linked to chemoresistance and poor prognosis in patients with colon cancer [^40^](#_ENREF_40)^,^[^41^](#_ENREF_41). In this current work, TRV130 was found to downregulate Cyclin D1 and Bcl-2 protein expression in a concentration-dependent manner, while significantly activating Caspase 3. This molecular change closely aligns with its phenotypic effects of inhibiting proliferation and promoting apoptosis. This mechanism is consistent with that of CDK4/6 inhibitors (e.g., palbociclib), which exert their effects by inhibiting the Cyclin D-CDK4/6 complex [^42^](#_ENREF_42)^,^[^43^](#_ENREF_43). However, as a non-kinase inhibitor, the specificity and off-target effects of TRV130 require further investigation.

Aberrant activation of the Hh pathway can drive the expansion of cancer stem cells and the maintenance of malignant phenotypes by remodeling embryonic proliferation signals, and it has been confirmed to be strongly related to the occurrence, progression, chemoresistance, and metastasis of colon cancer [^44^](#_ENREF_44)^,^[^45^](#_ENREF_45). As an upstream negative regulator of the pathway, PTCH1 normally inhibits SMO; loss of PTCH1 function or downregulated PTCH1 expression relieves this inhibition, thereby activating the downstream transcription factor GLI1 [^46^](#_ENREF_46)^,^[^47^](#_ENREF_47). GLI1 can bind to the promoter regions of target genes, driving their expression and promoting tumor progression [^48^](#_ENREF_48). In this study, TRV130 downregulated the protein expression of PTCH1 and GLI1 in colon cancer cells, with effects consistent with those of the positive control drug Cyc. As the effector terminal of the pathway, the functional activation of GLI1 is a prerequisite for the metastasis of colon cancer. Following the downregulation of GLI1 by TRV130, Cyclin D1 and Bcl-2 levels were concomitantly decreased, and cleaved caspase 3 levels were increased, suggesting that the inhibitory effect of TRV130 on the Hh pathway could be translated into anti-tumor phenotypes through downstream effector molecules. The consistency between the anti-tumor effect of TRV130 and the downregulation of pathway molecules as well as the regulation of proliferation- and apoptosis-related proteins in *in vivo* experiments strongly suggests that Hh pathway inhibition is a core mechanism underlying the anti-colon cancer activity of TRV130.

Currently, clinically available Hh pathway inhibitors (e.g., vismodegib) can inhibit tumor growth, but their clinical application is limited by the high risk of acquired drug resistance (resulting from their targeting of SMO) and severe adverse reactions such as skin toxicity and dysgeusia [^49^](#_ENREF_49)^,^[^50^](#_ENREF_50). Although small-molecule drugs that directly target GLI1 can circumvent SMO-mediated resistance, their clinical translation is hindered by high off-target risks because the zinc finger structure of GLI1 shares high structural homology with that of other transcription factors, and no such drugs have entered Phase III clinical trials to date [^51^](#_ENREF_51). Notably, targeted regulation of distinct molecular pathways is effective for colon cancer treatment, as demonstrated by Gelsolin’s inhibition of colon cancer proliferation via the TNFR2/CASP10 death receptor pathway [^52^](#_ENREF_52). This supports TRV130’s value as a clinically approved analgesic with a unique Hh pathway-targeting mechanism, offering a novel translational alternative.

As a clinically approved biased μ-opioid receptor agonist for the treatment of moderate to severe acute pain, TRV130 has well-validated pharmacokinetic stability and clinical safety [^53^](#_ENREF_53), which endows it with unique translational advantages in colon cancer treatment. Compared with de novo developed targeted drugs, the “known safety profile” of TRV130 can significantly shorten the preclinical toxicity evaluation cycle and reduce late-stage development risks. This holds important practical significance for colon cancer patients with concurrent cancer pain, as TRV130 is expected to address the dual needs of “pain management and tumor treatment” with a single agent, thereby reducing adverse reactions associated with polypharmacy.

Notably, previous studies have suggested that opioid drugs may influence tumor progression by regulating the tumor microenvironment [^54^](#_ENREF_54), but this study did not investigate whether TRV130 mediates the inhibitory effect on the Hh pathway through the μ-opioid receptor. Future studies should clarify the association between the “TRV130-μ-opioid receptor-Hh pathway” components via receptor knockdown/knockout experiments to further refine the molecular mechanism network. Additionally, the subcutaneous tumor xenograft model used in this study did not simulate clinically common scenarios such as peritoneal, hepatic, or pulmonary metastasis of colon cancer, nor did it include clinical samples to verify correlation between Hh pathway molecule expression and TRV130 responsiveness. These limitations should be addressed in subsequent studies through orthotopic metastasis models and clinical specimen analysis.

In summary, TRV130 can inhibit the Hh signaling pathway, thereby suppressing colon cancer cell proliferation, inducing apoptosis, blocking invasion and metastasis, and effectively inhibiting tumor growth in nude mouse tumor xenograft models. This finding not only fills the gap in research on the anti-tumor effects of TRV130 but also addresses the target limitations of traditional Hh inhibitors, providing a novel “safe, controllable, and multi-effect synergistic” candidate drug for colon cancer treatment. In the future, combination therapy experiments using TRV130 with existing chemotherapeutic drugs can be further conducted to explore its potential in reversing drug resistance. Moreover, verifying its association with patient prognosis through clinical samples will lay the foundation for advancing its entry into clinical research for colon cancer.

**Declarations
Ethics approval and consent to participate**

The present study was approved by the ethical review committee of Quanzhou first hospital Affiliated to Fujian Medical University. Written informed consent was obtained from all enrolled patients.

**Consent for publication**

Patients agree to participate in this work

**Availability of data and materials**

Data sharing not applicable to this article as no datasets were generated or analysed during the current study.

**Acknowledgement**

None.

**Funding**

None

**References**

1. Morgan E, Arnold M. Global burden of colorectal cancer in 2020 and 2040: incidence and mortality estimates from GLOBOCAN. *Gut*. 2023;72:338-344.

2. Balboa-Barreiro V, Pértega-Díaz S, García-Rodríguez T, et al. Colorectal cancer recurrence and its impact on survival after curative surgery: An analysis based on multistate models. *Digest Liver Dis*. 2024;56:1229-1236.

3. Lv P, Ren B, Cheng G. Ginsenoside Rh3 suppresses cell proliferation, metastasis, and oxidative stress of colorectal cancer via targeting HSP90AA1. *Lett Drug Design Discov* 2025;22:100082.

4. Chang H, Yu X, Xiao WW, et al. Neoadjuvant chemoradiotherapy followed by surgery in patients with unresectable locally advanced colon cancer: a prospective observational study. *Onco Targets Ther*. 2018;11:409-418.

5. Xie Y-H, Chen Y-X, Fang J-Y. Comprehensive review of targeted therapy for colorectal cancer. *Signal Transduct Tar*. 2020;5:22.

6. Singh M, Morris VK, Bandey IN, Hong DS, Kopetz S. Advancements in combining targeted therapy and immunotherapy for colorectal cancer. *Trends Cancer*. 2024;10:598-609.

7. Wu CC, Sytwu HK, Lu KC, Lin YF. Role of T cells in type 2 diabetic nephropathy. *Exp Diabetes Res*. 2011;2011:514738.

8. Alasgarova N, Baran, A., Yıldıztekin, M., Ganbarov, D., Babayeva, S., Güneş, Z., Evcil, M. Synthesis of Plant-Derived Selenium Nanoparticles from Lankaran-Astara Tea (Camellia sinensis L.) Plant and Evaluation of their Activities on Different Enzymes. *Adv Biol Earth Sci*. 2025;10:262-269.

9. Arthanari SK, Vanitha J, Ganesh M, Venkateshwaran K, Clercq D. Evaluation of antiviral and cytotoxic activities of methanolic extract of S. grandiflora (Fabaceae) flowers. *Asian Pac J Trop Bio* 2012;2:S855-S858.

10. Evcil M, Kurt B, Baran A, Mouhoub A, Karakaplan M. Development, Characterization and Application of Chitosan-Based Formulation Incorporating Crataegus orientalis Extract for Food Conservation. *Adv Biol Earth Sci* 2025;10:208-225.

11. Montazersaheb S, Eftekhari A, Shafaroodi A, et al. Green-synthesized silver nanoparticles from peel extract of pumpkin as a potent radiosensitizer against triple-negative breast cancer (TNBC). *Cancer Nanotechnol*. 2024;15:47.

12. Öziç C, Ertaş E, Baran MF, et al. Synthesis and characterization of activated carbon-supported magnetic nanocomposite (MNPs-OLAC) obtained from okra leaves as a nanocarrier for targeted delivery of morin hydrate. *Front Pharmacol*. 2024;15:1482130.

13. Baran A, Ertaş E, Baran MF. Green-Synthesized Characterization, Antioxidant and Antibacterial Applications of CtAC/MNPs-Ag Nanocomposites. *Pharmaceuticals (Basel)*. 2024;17:772.

14. Yaman U, Ince-Erguc, E., Ozturk, I., Okudan, E. S. Evaluation of Cytotoxic and Antimicrobial Activities of Methanolic Extracts from Cystoseira foeniculacea and Sargassum vulgare. *Adv Biol Earth Sci*. 2025;10:347-358.

15. Tumur S, Cihan, A., & Zahir Duz, M. Toxic Elements (As3+, Pb2+, Hg2+, Cd2+) In Wastewater: A Case Study On Environmental Threats And Health Risks. *Ad Biol Earth Sci* 2025;10:289-302.

16. Lorente JS, Sokolov AV, Ferguson G, et al. GPCR drug discovery: new agents, targets and indications. *Nat Rev Drug Discov*. 2025;24:458-479.

17. Zhao J, DiGiacomo V, Ferreras-Gutierrez M. Small-molecule targeting of GPCR-independent noncanonical G-protein signaling in cancer. *Proc Natl Acad Sci USA*. 2023;120:e2213140120.

18. Gareev I, Pavlov, V., Eyvazova, K., Mashkin, A., Iessa Obeid, A. A.Yang, L. Harnessing Adrenergic Receptor Pathways in Gliomas: From Tumor Biology to Targeted Therapies. *Adv Biol Earth Sci* 2025;10:245-261.

19. Yi K, Sun W. Overview and Prospects of the Clinical Application of Oliceridine. *Drug Des Devel Ther*. 2025;19:5415-5430.

20. Fossler MJ, Sadler BM, Farrell C, et al. Oliceridine (TRV130), a Novel G Protein-Biased Ligand at the μ-Opioid Receptor, Demonstrates a Predictable Relationship Between Plasma Concentrations and Pain Relief. I: Development of a Pharmacokinetic/Pharmacodynamic Model. *J Clin Pharmacol*. 2018;58:750-761.

21. Wang C, Liu L, Bai X. Global Trends in Oliceridine (TRV130) Research from 2013 to 2024: A Bibliometrics and Knowledge Graph Analysis. *Drug Des Devel Ther*. 2024;18:4681-4692.

22. Jing J, Wu Z, Wang J, et al. Hedgehog signaling in tissue homeostasis, cancers, and targeted therapies. *Signal Transduct Target Ther*. 2023;8:315.

23. Salaritabar A, Berindan-Neagoe I, Darvish B, et al. Targeting Hedgehog signaling pathway: Paving the road for cancer therapy. *Pharmacol Res*. 2019;141:466-480.

24. Cong G, Zhu X, Chen XR, Chen H, Chong W. Mechanisms and therapeutic potential of the hedgehog signaling pathway in cancer. *Cell Death Discov*. 2025;11:40.

25. Qian H, Cao P, Hu M, et al. Inhibition of tetrameric Patched1 by Sonic Hedgehog through an asymmetric paradigm. *Nat Commun*. 2019;10:2320.

26. Zhang L, Zhang Y, Li K, Xue S. Hedgehog signaling and the glioma-associated oncogene in cancer radioresistance. *Front Cell Dev Biol*. 2023;11:1257173.

27. Sriramulu S, Malayaperumal S, Banerjee A, et al. AEG-1 as a Novel Therapeutic Target in Colon Cancer: A Study from Silencing AEG-1 in BALB/c Mice to Large Data Analysis. *Curr Gene Ther*. 2024;24:307-320.

28. Jiang J. Hedgehog signaling mechanism and role in cancer. *Semin Cancer Biol*. 2022;85:107-122.

29. Pang F, Yang P, Wang T, et al. Comprehensive Analysis of Alternative Polyadenylation Events Associated with the Tumor Immune Microenvironment in Colon Adenocarcinoma. *Curr Genomics*. 2023;24:48-61.

30. Xi Y, Xu P. Global colorectal cancer burden in 2020 and projections to 2040. *Transl Oncol*. 2021;14:101174.

31. Zhan Z, Chen B, Lin W, et al. Rising Burden of Colon and Rectum Cancer in China: An Analysis of Trends, Gender Disparities, and Projections to 2030. *Ann Surg Oncol*. 2025;32:3361-3371.

32. Wu H, Chen W, Chen Z, Li X, Wang M. Novel tumor therapy strategies targeting endoplasmic reticulum-mitochondria signal pathways. *Ageing Res Rev*. 2023;88:101951.

33. Li Y, Liu F, Cai Q, et al. Invasion and metastasis in cancer: molecular insights and therapeutic targets. *Signal Transduct Tar*. 2025;10:57.

34. Wang P, Hua X, Sun Y, et al. Loss of haspin suppresses cancer cell proliferation by interfering with cell cycle progression at multiple stages. *FASEB J*. 2021;35:e21923.

35. Glaviano A, Singh SK, Lee EHC, et al. Cell cycle dysregulation in cancer. *Pharmacol Rev*. 2025;77:100030.

36. Mosadegh M, Noori Goodarzi N, Erfani Y. A Comprehensive Insight into Apoptosis: Molecular Mechanisms, Signaling Pathways, and Modulating Therapeutics. *Cancer Invest*. 2025;43:33-58.

37. Kouroshnia A, Zeinali S, Irani S, Sadeghi A. Induction of apoptosis and cell cycle arrest in colorectal cancer cells by novel anticancer metabolites of Streptomyces sp. 801. *Cancer Cell Int*. 2022;22:235.

38. Jun SY, Kim J, Yoon N, Maeng LS, Byun JH. Prognostic Potential of Cyclin D1 Expression in Colorectal Cancer. *J Clin Med*. 2023;12:572.

39. Hume S, Dianov GL, Ramadan K. A unified model for the G1/S cell cycle transition. *Nucleic Acids Res*. 2020;48:12483-12501.

40. Wang L, Xi C, Liu R, et al. Dual targeting of Mcl-1 and Bcl-2 to overcome chemoresistance in cervical and colon cancer. *Anticancer Drug*. 2024;35:219-226.

41. Vogler M, Braun Y, Smith VM, et al. The BCL2 family: from apoptosis mechanisms to new advances in targeted therapy. *Signal Transduct Tar*. 2025;10:91.

42. Adon T, Shanmugarajan D, Kumar HY. CDK4/6 inhibitors: a brief overview and prospective research directions. *RSC Adv*. 2021;11:29227-29246.

43. Braal CL, Jongbloed EM, Wilting SM, et al. Inhibiting CDK4/6 in Breast Cancer with Palbociclib, Ribociclib, and Abemaciclib: Similarities and Differences. *Drugs*. 2021;81:317-331.

44. Omar A, Ruff P, Penny C. Inhibition of the Sonic Hedgehog Pathway using Small Molecule Inhibitors: Targeting Colon Cancer Stem Cells. *Curr Cancer Ther Rev*. 2023;19:138-155.

45. Sari IN, Phi LTH, Jun N, et al. Hedgehog Signaling in Cancer: A Prospective Therapeutic Target for Eradicating Cancer Stem Cells. *Cells*. 2018;7:208.

46. Kinnebrew M, Woolley RE, Ansell TB, et al. Patched 1 regulates Smoothened by controlling sterol binding to its extracellular cysteine-rich domain. *Sci Adv*. 8:5563.

47. Kovachka S, Malloci G, Simsir M, et al. Inhibition of the drug efflux activity of Ptch1 as a promising strategy to overcome chemotherapy resistance in cancer cells. *Eur J Med Chem*. 2022;236:114306.

48. Sigafoos AN, Paradise BD, Fernandez-Zapico ME. Hedgehog/GLI Signaling Pathway: Transduction, Regulation, and Implications for Disease. *Cancers (Basel)*. 2021;13:

49. Wang B, Zhao K, Xian N, et al. Adverse events associated with vismodegib: insights from a real-world pharmacovigilance study using the FAERS database. *Front Pharmacol*. 2025;16:1497708.

50. Liao S, Floyd C, Verratti N, Leung L, Wu C. Analysis of vismodegib resistance in D473G and W535L mutants of SMO receptor and design of novel drug derivatives using molecular dynamics simulations. *Life Sci*. 2020;244:117302.

51. Maresca L, Crivaro E, Migliorini F, et al. Targeting GLI1 and GLI2 with small molecule inhibitors to suppress GLI-dependent transcription and tumor growth. *Pharmacol Res*. 2023;195:106858.

52. Wang Z, Song W. Gelsolin Inhibits the Proliferation of Colon Cancer Cells by Enhancing the Expression of TNFR2/CASP10 as a Death Receptor Pathway. *Protein Pept Lett*. 2023;30:214-220.

53. Miyano K, Manabe S, Komatsu A, et al. The G Protein Signal-Biased Compound TRV130; Structures, Its Site of Action and Clinical Studies. *Curr Top Med Chem*. 2020;20:2822-2829.

54. Wang R, Li S, Wang B, Wang G, Zheng H. Impact of opioids and mu-opioid receptors on oncologic metastasis. *Am J Cancer Res*. 2024;14:4236-4247.

**Figure legends**

**Fig. 1 TRV130 represses proliferation of colon cancer cells.** (A) Caco-2 and HCT116 cells were treated with TRV130 at varying concentrations (2.5, 5, 10, 20, 40, 80, and 160 μM) for 24 hours, and cell viability was measured by MTT assay. (B-C) Caco-2 and HCT116 cells were treated with different concentrations of TRV130 (10, 20, and 40 μM). (B) EdU incorporation assay was used to evaluate cell proliferation in Caco-2 and HCT116 cells (Scale bar = 100 μm). (C) Colony formation assay was performed to assess the clonogenic potential of Caco-2 and HCT116 cells. * *P*<0.05, *** *P* < 0.001, ns, not significant.

**Fig. 2 TRV130 induces apoptosis in colon cancer cells in a concentration-dependent manner.** (A) Apoptosis of Caco-2 and HCT116 colon cancer cells treated with TRV130 (10, 20, and 40 μM) was analyzed by Annexin V-FITC/PI flow cytometry, and the apoptosis rate was quantified.(B) TUNEL staining was used to detect apoptotic cells in Caco-2 and HCT116 colon cancer cells after TRV130 treatment (10, 20, and 40 μM) (Scale bar = 100 μm). * *P*<0.05, ** *P*<0.01, *** *P* < 0.001.

**Fig. 3** **TRV130 suppresses migration and invasion of colon cancer cells.** Caco-2 and HCT116 cells were treated with 10, 20, and 40 μM TRV130. (A-B) Transwell assays were performed to assess the migratory and invasive capacity of Caco-2 and HCT116 cells (Scale bar = 50 μm). (C) Wound healing assays were used to detect the migratory potential of Caco-2 and HCT116 cells; representative images at 0 hour and 24 hours were captured, and the wound closure percentage was quantified (Scale bar = 100 μm). * *P*<0.05, ** *P*<0.01, *** *P*<0.001, ns, not significant.

**Fig. 4 TRV130 modulates expression of cell proliferation- and apoptosis-related proteins in colon cancer cells.** Caco-2 and HCT116 cells were treated with TRV130 (10, 20, and 40 μM). (A) Western blot analysis of Cyclin D1, Bcl-2, total-caspase 3, and C-caspase 3 expression levels in Caco-2 cells. (B) Western blot analysis of Cyclin D1, Bcl-2, total-caspase 3, and C-caspase 3 levels in HCT116 cells. * *P*<0.05, ** *P*<0.01, *** *P*<0.001.

**Fig. 5 TRV130 inhibits the Hh signaling pathway in colon cancer cells.** Caco-2 cells and HCT116 cells were treated TRV130 (10, 20, and 40 μM) or Cyc (5 μM) (A-B) Western blot analysis of GLI1 and PTCH1 (key Hh pathway proteins) expression levels in Caco-2 and HCT116 cells. * *P*<0.05, ** *P*<0.01, *** *P*<0.001.

**Fig. 6 TRV130 constrains tumor growth and regulates Hh pathway and apoptosis-related proteins in HCT116 xenograft mice.** Mice were divided into 4 groups: Saline, TRV130 (1.18 mg/kg), TRV130 (3.6 mg/kg), and Cyc (30 mg/kg). (A) Tumor volume was measured every 5 days in xenograft mice.(B) Representative images of excised tumors (left) and quantitative analysis of tumor weight (right) at the endpoint. (C) IHC staining of GLI1 and PTCH1 in tumor tissues; scale bar = 50 μm. (D) Western blot analysis of GLI1, PTCH1, Cyclin D1, Bcl-2, total-caspase 3, and C-caspase 3 in tumor tissues. ** *P*<0.01, *** *P*<0.001, ns, not significant.

**Fig. S1 TRV130 dose-dependently upregulates the expression of apoptotic pathway-related proteins in colon cancer cells.** Caco-2 and HCT116 cells were treated with TRV130 (10, 20, and 40 μM). (A-B) Western blot analysis of Bax, Bim, and FasL expression levels in Caco-2 and HCT116 cells. * *P*<0.05, ** *P*<0.01, *** *P*<0.001.
